# FOS-driven inflammatory CAFs promote colorectal cancer liver metastasis via the SFRP1-FGFR2-HIF1 axis

**DOI:** 10.7150/thno.111625

**Published:** 2025-03-21

**Authors:** Long Liu, Yuhao Ba, Shuaixi Yang, Aning Zuo, Shutong Liu, Yuyuan Zhang, Shuqin Xu, Siyuan Weng, Benyu Liu, Peng Luo, Quan Cheng, Jinhai Deng, Hui Xu, Yukang Chen, Chuhan Zhang, Xing Zhou, Yuqing Ren, Xinwei Han, Zhenyu Hou, Zaoqu Liu

**Affiliations:** 1 Department of Interventional Radiology, The First Affiliated Hospital of Zhengzhou University, Zhengzhou, Henan, 450052, China.; 2 Department of hepatobiliary surgery, the First Affiliated Hospital of Xi'an Jiaotong University, Xi'an, Shanxi, 710061, China.; 3 Interventional Institute of Zhengzhou University, Zhengzhou, Henan, 450052, China.; 4 Interventional Treatment and Clinical Research Center of Henan Province, Zhengzhou, Henan, 450052, China.; 5 Department of Colorectal Surgery, The First Affiliated Hospital of Zhengzhou University, Zhengzhou, 450000, Henan, China.; 6 National Cancer Center/National Clinical Research Center for Cancer/Cancer Hospital, Chinese Academy of Medical Sciences and Peking Union Medical College, Beijing, 100021, China.; 7 Tianjian Laboratory of Advanced Biomedical Sciences, Academy of Medical Sciences, Zhengzhou University, Zhengzhou, China.; 8 The Department of Oncology, Zhujiang Hospital, Southern Medical University, Guangzhou, China.; 9 Department of Neurosurgery, Xiangya Hospital, Central South University, Changsha, Hunan, China.; 10 Richard Dimbleby Department of Cancer Research, Comprehensive Cancer Centre, Kings College London, London, United Kingdom.; 11 Department of Oncology, The First Affiliated Hospital of Zhengzhou University, Zhengzhou, Henan, 450052, China.; 12 Department of Pediatric Surgery, The First Affiliated Hospital of Zhengzhou University, Zhengzhou, Henan, 450052, China.; 13 Department of Respiratory and Critical Care Medicine, The First Affiliated Hospital of Zhengzhou University, Zhengzhou, Henan, 450052, China.; 14 Department of General Surgery, Qilu Hospital of Shandong University, Jinan, Shandong, 250012, China.; 15 Institute of Basic Medical Sciences, Chinese Academy of Medical Sciences and Peking Union Medical College, Beijing, 100730, China.

**Keywords:** Colorectal cancer liver metastasis, Single-cell RNA sequencing, Cancer-associated fibroblasts, SFRP1, Therapeutic target

## Abstract

**Rationale:** Cancer-associated fibroblasts (CAFs) exhibit diverse functions, yet their roles in colorectal cancer liver metastasis (CRLM) remain poorly understood.

**Methods:** Through integrated analysis of single-cell RNA sequencing and spatial transcriptomics from colorectal cancer patients (CRCP: non-metastatic primary tumors; CRCM: metastatic primary tumors with liver metastases), combined with *in vitro* and *in vivo* models to investigate the role of CAFs in CRLM. *In vitro* experiments included six groups to reveal the role of SFRP1-producing CAFs, comprising PBS (control) and recombinant human SFRP1 (rhSFRP1) treated SW480 cells, PBS (control) and recombinant mouse SFRP1 (rmSFRP1) treated CT26 cells, and conditioned medium (CM) derived from CAF-NC and CAF-Sfrp1 treated CT26 cells. Preclinical models were further employed to elucidate the role of SFRP1 in CRLM. Subcutaneous xenografts models were constructed from PBS (control) and rhSFRP1 treated SW480 cells. For orthotopic tumor metastasis models, CT26 cells were pre-cultured with CAF-NC or CAF-Sfrp1 and then orthotopically injected into BALB/c mice.

**Results:** We identified an inflammatory CAF subtype (CFD^+^ iCAFs) associated with poor clinical outcomes, advanced staging, and metastasis. Transcriptional regulation analysis revealed FOS-mediated differentiation of CFD^+^ iCAFs drives SFRP1 overexpression. *In vitro* and *in vivo* experiments confirmed that SFRP1-producing CAFs promote tumor stemness and epithelial-mesenchymal transition (EMT). Mechanistically, SFRP1 from CFD^+^ iCAFs binds FGFR2, activating the HIF1 signaling pathway to enhance tumor stemness, EMT, and CRLM progression.

**Conclusion:** This study highlights CFD^+^ iCAFs as key regulators of tumor-stromal interactions and identifies SFRP1 as a potential therapeutic target in CRLM.

## Introduction

Colorectal cancer (CRC) is the second most lethal tumor globally, with metastasis responsible for 90% of CRC mortality 1. The colorectal cancer liver metastasis (CRLM) is a dynamic process modulated by cell interactions within tumor microenvironment (TME) 2. Cancer-associated fibroblasts (CAFs) are ubiquitous stromal cells within the TME, exerting diverse and critical functions in cancer metastasis, including matrix production and remodeling, angiogenesis 3. Specifically, CAFs enhance extracellular matrix remodeling via transforming growth factor beta (TGF-β) signaling pathway, promoting cancer metastasis 4, 5. They also release VEGFA and matrix metalloproteinases (MMPs) to facilitate angiogenesis, thereby providing pathways for cancer cells to metastasize through intravasation and extravasation 4. Recently, single-cell RNA sequencing (scRNA-seq) has brought advancements in unveiling remarkable heterogeneity of CAFs 6. With the advent of high-resolution scRNA-seq, distinct CAFs phenotypic subtypes are revealed, such as matrix CAFs (mCAFs) supporting matrix remodeling, inflammatory CAFs (iCAFs) secreting cytokines, antigen presenting CAFs (apCAFs) promoting antigen presentation, and vascular CAFs (vCAFs) driving angiogenesis 7, 8. The high degree of heterogeneity among CAFs indicates that distinct subtypes may exert unique biological effects in CRLM. Currently, the precise identification of which CAF subtypes drive CRLM and the underlying mechanisms by which they promote this process remain largely unexplored.

Emerging research has suggested that CAFs modulate TME promoting metastasis through interactions with other cells 9, 10. CAFs interact directly with cancer cells to drive metastatic potential via secreting hepatocyte growth factor (HGF) 9. The crosstalk between CAFs and immune cells promote an immunosuppressive TME, fostering the formation of a pre-metastatic ecological niche, and ultimately driving tumor progression and metastasis 10. These interactions are usually mediated by a variety of mechanisms 11, including the secretion of growth factors, cytokines, chemokines, extensive reciprocal cell interactions, and intricate signaling conduction, yet many aspects of these processes remain incompletely understood in CRLM. Notably, the plasticity of CAFs allows them to adapt and evolve dynamically within TME during tumor metastasis 12. The transcription factors (TFs) are key for CAFs plasticity, which govern the activation and functional maintenance of CAFs, and regulate cell reciprocal interactions with tumor cells 13. The scRNA-seq has partially elucidated the heterogeneity of CAFs, while the mechanisms regulating CAFs plasticity and driving CAFs transformation into a specific metastasis-associated subtypes, remain poorly understood. Elucidating the complex interplay within TME and revealing the plasticity of CAF subtypes might unlock novel approaches to disrupt metastatic cascades. Addressing these gaps, including identification of specific pro-metastatic CAF subtypes, the plasticity of CAFs, and the detailed mechanisms between cells crosstalk, which is essential for a comprehensive understanding of CAFs in CRLM and for the development of targeted therapeutic strategies.

In this study, we conducted a comprehensive investigation on the role of CAFs and their interactions with cancer cells in CRLM. Our findings identified a specific subpopulation CFD^+^ iCAFs that display a pro-metastatic effect in CRC. Trajectory analysis, ChIP assay, and luciferase reporter assay demonstrated transcription factor FOS drives the formation of CFD^+^ iCAFs and promotes the expression of effector molecule SFRP1. The pro-metastatic effect was based on the interaction between secretory protein SFRP1 and FGFR2 receptor activates HIF1 signaling pathway, uncovering SFRP1-FGFR2-HIF1 signaling axis in CRLM. Overall, our findings demonstrate that FOS drives the formation of pro-metastatic CFD^+^ iCAFs and secretory protein SFRP1 derived from CAFs promotes CRLM through SFRP1-FGFR2-HIF1 signaling axis.

## Methods

### Single cell and spatial transcriptomics analysis

#### Data acquisition and processing of single cell RNA sequencing

Single cell RNA sequencing (scRNA-seq) data were collected and enrolled to investigate colorectal cancer liver metastasis (CRLM), including GSE144735 14, GSE178318 15, and GSE200997 16. The scRNA-seq data were processed following *Seurat* package pipeline and quality control standards were conducted in the following manner. 1) To ensure data consistency and comparability, we only retrieved primary CRC tissue samples with liver metastasis (CRCM) and those without liver metastasis (CRCP), encompassing 45,141 cells. 2) Doublets were instances where two cells were captured together and mistaken for a single cell, distorting the interpretation of cell types or states. Probable doublets were predicted and removed using *DoubletFinder* R package. Low quality cells characterized by low gene detection rates or elevated mitochondrial gene expression due to stress or damage, can introduce noise into the data. Here, low quality cells with detected gene number fewer than 500 and mitochondrial gene contents more than 20% were removed for subsequent analysis. 3) Data normalization was implemented using NormalizeData function and top 2000 highly variable genes were selected by variance stabilizing transformation (VST) method. The unwanted sources of variation were regressed out using ScaleData function. Dimensionality reduction was performed via principal component analysis (PCA) and the suitable principal component (PCs) were provided by Elbowplot with the value of 30. 4) Batch effects were corrected among samples using Harmony algorithm 17. This method could identify and remove batch-specific variations while preserving biological differences.

Cell clustering was achieved using FindNeighbors and FindClusters function. The Uniform Manifold Approximation and Projection (UMAP) was employed to visualize the clustering. Differentially expressed genes (DEGs) of each clustering were identified using FindAllMarkers function. The cell clustering was initially labeled and annotated based on DEGs and known markers, such as T cells (CD2, CD3D, CD3E, TRAC); NK cells (NKG7, KLRD1, PRF1); Epithelial cells (EPCAM, KRT18, KRT8); Myeloid cells (CD14, CD68, LYZ); CAFs (COL1A1, COL3A1, DCN); Endothelial cells (VWF, PECAM1, CLDN5, ENG); Mast cells (TPSAB1, TPSB2, MS4A2); Plasma cells (IGHA1, MZB1); B cells (CD79A, CD79B, MS4A1). The *InferCNV* package 18 was further performed to identify malignant cells and non-malignant cells among epithelial cells according to genomic copy-number variations.

#### Cell classification for CAFs

To further reveal the heterogeneity of CAFs at finer levels, CAFs subpopulations were re-clustered using *Seurat* package. The LogNormalize method and VST method were used to perform data normalization and identify highly variable genes. PCA was utilized to dimensionality reduction and 20 principal components were retained. Next, the FindNeighbors function was employed to construct a shared nearest neighbor and FindClusters function was applied to identify cell clusters using Louvain algorithm. The resulting subclusters of CAFs subpopulations were visualized using UMAP. To annotate these subclusters, we proceed according to the following standards. 1) The FindMarkers function was used to identify DEGs of each subcluster. These genes were then cross-referenced with classical marker molecules and previous studies 3, 4, 8, such as HLA-DQA1, CFD, POSTN, and MMP3, performing initially biological identities to each subcluster. 2) The AddModuleScore function was employed to score gene signatures and verify initial identities, including mCAFs, iCAFs, apCAFs, and vCAFs. 3) Functional enrichments were used to reveal key biological processes and signaling pathways associated with each subcluster, providing further validation for CAFs classification.

#### Functional enrichment analysis

The gene sets were obtained from MSigDB resource, including c2.cp.kegg.v7.5.1, c5.go.v7.5.1, h.all.v7.5.1. Gene Set Enrichment Analysis (GSEA) was performed to identify distinct biological characteristic between different groups 19. For upregulated and downregulated genes, the over-representation analysis (ORA) was conducted to GO and KEGG enrichment. Metascape was used to connect similar pathways into a network, presenting the enrichment results. For specific pathway activity, the single-sample gene set enrichment analysis (ssGSEA) 20 was used to calculate score.

#### Cell-cell communications and proportional changes

CellChat was usually used to quantitatively infer cell-cell communication networks from scRNA-seq data 21. The cell composition was calculated and further presented using sankey diagram. The Ro/e was calculated following Observed/Expected, the ratio of observed over expected cell numbers in a given cell type 22.

#### Trajectory inference

The *Monocle2* package 23 was applied to explore potential cell lineage trajectory among CAFs. DEGs were identified among distinct state of CAFs and top 100 significant genes ranked with q value were displayed along the pseudotime trajectory. The *CytoTRACE* package 24 was used to infer cell differentiation states of CAFs.

#### Identification of single-cell regulatory network

The *SCENIC* package 25 was employed to identify the important transcription factors (TFs) in CFD^+^ iCAFs. Single cell expression matrix was served as input data, and latest motif dataset was employed to construct regulons for TFs. Then, the GENIE3 algorithm was utilized to generate gene regulatory networks. The AUCell algorithm was applied to quantify regulon activity and regulon specificity score (RSS) was further calculated for cell-type specificity 26. To evaluate the importance of FOS, we also retrieved FOS CHIP-sequencing data, including GSM2825448, GSM2825449, GSM2827569, GSM2827570, GSM7247377, and GSM7247378. Based on hg38 database, motifs density was scored in gene promoter regions with 3,000 bp around the transcription start site (TSS) by *ChIPseeker* and *ggChIPvis* package 27.

#### Spatial transcriptomics analysis

To explore cell-cell and ligand-receptor colocalization, the 10X Visium Spatial transcriptome data was enrolled. The 10X Visium Spatial transcriptome data were retrieved from the scCRLM atlas (http://www.cancerdiversity.asia/scCRLM/). These data originated from 4 primary CRC and matched liver metastasis patients and contained 31,203 spots within tissue regions. We performed quality control as the following criteria: spots with number below 300, and mitochondrial gene numbers exceeding 30%. The AddModuleScore function was employed to score gene signatures to present cell-cell interactions. The *SpaGene* package 28 was used to infer ligand-receptor interactions through their colocalization.

### *In vitro* functional experiments

#### Isolation of primary cancer-associated fibroblasts (CAFs)

Colon cancer tissues were harvested from BALB/c mice bearing orthotopic tumors generated. The tissues were washed thoroughly with 5× trypsin PBS (Servicebio, Wuhan, China). Clean tissues were cut into approximately 1 mm³ fragments and digested with 1 mg/mL type IV collagenase (Thermo Scientific, Shanghai, China) at 37 °C for 2 h. The digested suspension was centrifuged to discard the supernatant, filtered through a 200-mesh strainer, and the resulting cell pellet was resuspended in high-glucose DMEM supplemented with 10% fetal bovine serum.

#### Cell lines and cell culture

The SW480 and CT26 cell line were purchased from company (Priscilla, Hubei, China). Following initial culture of SW480 and CT26, cells were treated with complete medium containing 100 ng/mL rhSFRP1 or rmSFRP1. Alternatively, the medium of CT26 cells was replaced with 50% conditioned medium (CM) derived from mouse CAF-NC or CAF-Sfrp1. The CAF-NC pointed to control cells and the CAF-Sfrp1 referred to CAFs with stable overexpression of Sfrp1.

#### Lentiviral construction and generation of stable cell lines

The human shFGFR2 sequence was cloned into the lentiviral vector pLKO.1-EGFP-Puro and packaged into lentiviral particles. An empty vector was used as a control. Cells were infected with the lentiviral particles and selected with puromycin for 2 weeks to establish SW480 cells with stable knockdown of FGFR2. Similarly, stable knockdown of Fgfr2 in mouse cells was achieved using the same protocol as described above. The full-length mouse Sfrp1 gene was subcloned into the lentiviral vector pLent-EF1a-FH-CMV-RFP-Puro and packaged into pLent-Sfrp1 lentiviral particles. An empty vector (pLent-empty) served as a control. Cells were infected with either pLent-Sfrp1 or pLent-empty lentiviral particles and selected with puromycin for 2 weeks to generate CAFs with stable overexpression of Sfrp1 (CAF-Sfrp1) or control cells (CAF-NC). Similarly, the construction process of Fos stable overexpression CAFs (OE-Fos) was consistent with the above description.

#### Transient transfection of small interfering RNAs

Small interfering RNAs (siRNA) targeting Fos was purchased from OBiO Technology (Shanghai, China). Following the manufacturer's protocol, siRNA was transfected into CAFs using the Lipofectamine 3000 transfection reagent (Thermo Scientific, Shanghai, China).

#### Cellular behavior assays

CT26 and SW480 cells were seeded into 96-well ultra-low attachment plates (ThermoFisher, Waltham, USA) at a density of 2000 cells per well. The plates were placed on a constant-temperature shaker (37 °C, 120 rpm) for 3 h (CT26) or 6 h (SW480) to facilitate initial spheroid formation. After shaking, the plates were transferred to a 37 °C incubator for further culture. For colony formation, CRC cells were seeded into 6-well plates at a density of 2000 cells per well. The colonies were then fixed with 4% paraformaldehyde, stained with crystal violet, and photographed.

For migration assays, 2×10⁴ transfected cells were seeded into the upper chamber of Transwell inserts (Corning, New York, USA) without Matrigel, while 500 μL of complete medium was added to the lower chamber. For invasion assays, 4×10⁴ treated cells were seeded into Matrigel-coated upper chambers and incubated for 48 h. Migrated or invaded cells were fixed with 4% paraformaldehyde (Beyotime, Shanghai, China), stained with crystal violet (Solarbio, Beijing, China), and imaged under an optical microscope (Cewei, Shanghai, China). The number of cells was quantified using ImageJ software.

#### Chromatin immunoprecipitation (ChIP) and Luciferase reporter assay

ChIP assays were performed using the ChIP-IT Express Kit (Active Motif, Shanghai, China) according to the manufacturer's protocol. A portion of the chromatin was reserved as input control, and the rest was incubated with Fos or IgG antibodies and protein A magnetic beads overnight at 4 °C. Immunoprecipitated complexes were sequentially washed with ChIP wash buffers I and II. Crosslinking was reversed, and DNA was purified using phenol-chloroform extraction for subsequent PCR analysis. The Dual-Luciferase Reporter Assay Kit (Beyotime, Shanghai, China) was utilized to measure luciferase activity following the manufacturer's protocol. After transfection, cells were lysed directly in culture dishes using the provided lysis buffer, and the lysates were centrifuged at maximum speed for 1 min using a microcentrifuge. The relative luciferase activity was quantified with a Modulus™ TD20/20 Luminometer (Turner Biosystems, USA), and Renilla luciferase activity served as an internal reference to normalize transfection efficiency.

#### Western blot

Protein samples (25 µg each) were separated using 4-20% gradient SDS-PAGE gels and transferred to PVDF membranes (Millipore, Massachusetts, USA). Membranes were blocked with a rapid blocking solution for 20 min at room temperature, followed by incubation with primary antibodies. Detection was performed using HRP-conjugated secondary antibodies, and chemiluminescent signals were visualized using Super ECL Plus (UElandy, Suzhou, China) on a Tanon chemiluminescent imaging system (Tanon, Shanghai, China).

#### Immunoprecipitation-mass spectrometry (IP-MS)

Recombinant human SFRP1 protein was added to 7 × 10^6 SW480 cells and incubated for 48 h. Co-immunoprecipitation (Co-IP) was performed according to the manufacturer's instructions using the Pierce™ Crosslink Magnetic IP/Co-IP Kit (Thermo Fisher Scientific, 88805, Massachusetts, USA). Briefly, 10 μg of SFRP1-specific antibody or species-matched normal IgG was used. The samples were separated by SDS-PAGE on a 4-20% gel, which was then stained using a silver staining kit (Beyotime, P0017S, Shanghai, China). Protein bands of interest were excised and analyzed by mass spectrometry (MS) for protein identification by APTBIO (Shanghai, China).

#### Coimmunoprecipitation (Co-IP)

In the Co-IP experiment, the SFRP1 IP and IgG groups were derived from the IP-MS products mentioned above, with 20 μL of freshly prepared whole-cell lysate used as the input group. The samples were boiled at 100 °C for 10 min for Western blot analysis.

#### Organoid culture

The tissues were cut into 1-2 mm³ fragments and mechanically dissociated in organoid buffer for 2 min. After centrifugation, the pellet was digested in tissue dissociation solution (HonrayMed Biotechnology) at 37 °C for 30 min with gentle shaking. The digested mixture was passed through a 70 µm strainer to remove debris, centrifuged again, and the final cell pellet was resuspended in fresh organoid buffer.

The cell pellet was resuspended in 80% Matrigel, and 30 µL of the mixture was added into pre-warmed wells of a 24-well plate. The Matrigel was solidified by incubating the plate upright at 37 °C for 5 min, followed by an inverted incubation for 25 min. After solidification, 500-750 µL of organoid culture medium was added to each well. The cultures were maintained in a 37 °C incubator with 5% CO₂, and the medium was refreshed every 2-3 days. Growth and structural integrity were assessed by capturing images under an optical microscope and analyzing the organoid areas using ImageJ software.

#### Immunohistochemistry and immunofluorescence

For immunohistochemistry, sections were dewaxed in xylene, rehydrated through a graded alcohol series, and subjected to antigen retrieval in EDTA buffer (pH 8.0). Following antigen retrieval, sections were incubated with goat serum for 30 min at room temperature to block non-specific binding. The sections were then incubated with primary antibodies overnight at 4 °C. After washing, sections were stained with the corresponding secondary antibody for 1 h at room temperature. Finally, the sections were treated with DAB (Servicebio, Wuhan, China), counterstained with hematoxylin. Hematoxylin-eosin was performed using Hematoxylin-eosin (H&E) HD constant dye kit (Servicebio, Wuhan, China) according to the manufacturer's instructions. The bright-field images of tissue sections were obtained using an ECLIPSE E100 microscope (Nikon, Tokyo, Japan) and stained sections were scanned with a Pannoramic MIDI (3DHISTECH, Budapest, Hungary). Tissue microarray (HColA180Su16) contained 104 CRC patients with complete survival and stage clinical information were purchased from Superbiotek Pharmaceutical Technology (Shanghai, China).

For immunofluorescence, cells were seeded and treated with the specified conditions. Following treatment, cells were fixed with 4% paraformaldehyde at room temperature. After blocking with 5% bovine serum albumin (BSA) in PBS, cells were incubated with primary antibodies overnight at 4 °C. Following PBS washing, cells were incubated with the corresponding secondary antibody for 1 h at room temperature. Nuclei were stained with DAPI. Finally, the cells were mounted on slides and examined using a NIKON ECLIPSE C1 microscope (Nikon, Tokyo, Japan), with images captured on a Pannoramic MIDI scanner (3DHISTECH, Budapest, Hungary).

### *In vivo* functional validation experiments

#### Animal models

A randomization process was implemented to assign animals to different experimental groups. Meanwhile, double blind experimental design was performed during the experimental interventions and data analysis to prevent subjective bias. For every animal experiment, each experimental group and control group consisted of 6 mice. All animal procedures were reviewed and approved by the Institutional Animal Care and Use Committee of Zhengzhou University.

Four-week-old male athymic BALB/c nude mice and six-week-old male BALB/c and C57BL/6J mice were obtained from Vital River Laboratory Animal Technology (Beijing, China). For the subcutaneous model, each mouse was injected subcutaneously with 5×10⁶ SW480 or shFGFR2 SW480 cells suspended in 100 μL PBS, with 6 mice per group. Mice in the rhSFRP1 treatment group received intratumoral injections of rhSFRP1 every 4 days, while control mice were injected with an equal volume of PBS. For the inhibitor treatment, Echinomycin-treated mice received tail intravenous injection of 10 μg/kg Echinomycin in PBS (MCE, Shanghai, China) every 4 days, while the control group received PBS containing 10 μg/kg DMSO (MCE, Shanghai, China).

Six-week-old male BALB/c mice were used to establish the orthotopic colorectal cancer metastasis model. CT26 cells (1×10⁶) co-cultured with CAF-Sfrp1 or CAF-NC, as well as CT26 or shFgfr2 CT26 cells co-cultured with CAF-Sfrp1, were implanted into the cecal wall of anesthetized mice, with 6 mice per group. For the inhibitor treatment experiment, mice implanted with CT26 cells co-cultured with CAF-Sfrp1 received intraperitoneal injections every two days. The Echinomycin treatment group was administered PBS containing 10 μg/kg Echinomycin (MCE, Shanghai, China), while the control group received PBS containing 10 μg/kg DMSO (MCE, Shanghai, China).

Twelve 6-week-old male C57BL/6J mice were randomly divided into two groups (CAF-Sfrp1 and CAF-NC). A total of 1×10⁶ luciferase-labeled MC38 cells, pre-cultured with CAF-Sfrp1 or CAF-NC, were implanted into the cecal wall of each mouse. After 4 weeks, liver metastasis was evaluated using bioluminescence imaging. A stock solution of D-luciferin sodium salt (15 mg/mL) was prepared in PBS, and mice were intraperitoneally injected with D-luciferin (150 mg/kg) 30 min before imaging. Mice were anesthetized with 2% isoflurane during imaging, and the bioluminescent signals in the liver region were captured using the Tanon ABL X5 imaging system.

#### Patient-derived xenografts (PDXs) mouse model

Four-week-old male C-NSG mice were obtained from Cyagen Biosciences (Suzhou, China). Fresh CRC tissues from patients were cut into ~2 mm³ fragments and subcutaneously implanted into the right flank of C-NSG mice. Tumor growth was regularly monitored, and once tumors reached a suitable size, they were excised, sectioned into ~2 mm³ pieces, and implanted into a new group of mice to generate second-generation PDX tumors. When tumor volumes reached approximately 100 mm³, mice were randomly assigned to treatment groups. Body weight was recorded every 5 days to assess general health.

### Statistical analysis

The data processing, statistical analysis, and graphical plotting were performed in R (v 4.3.1). Comparation of two continuous variables were conducted by Wilcoxon rank-sum test or Student t test. The correlation was assessed using Spearman's correlation coefficients. For survival analysis, the optimal cut-off value was determined by *survminer* package. Kaplan-Meier analyses were performed, and log-rank test was tested using *survival* package. An alpha level of 0.05 was used for all statistical tests. The two-sided P value less than 0.05 was considered as statistically significant. Other detailed methods are available in the Supplementary Methods.

## Results

### Distinct CAF profiles in primary CRC with and without liver metastasis

**Figure 1:**
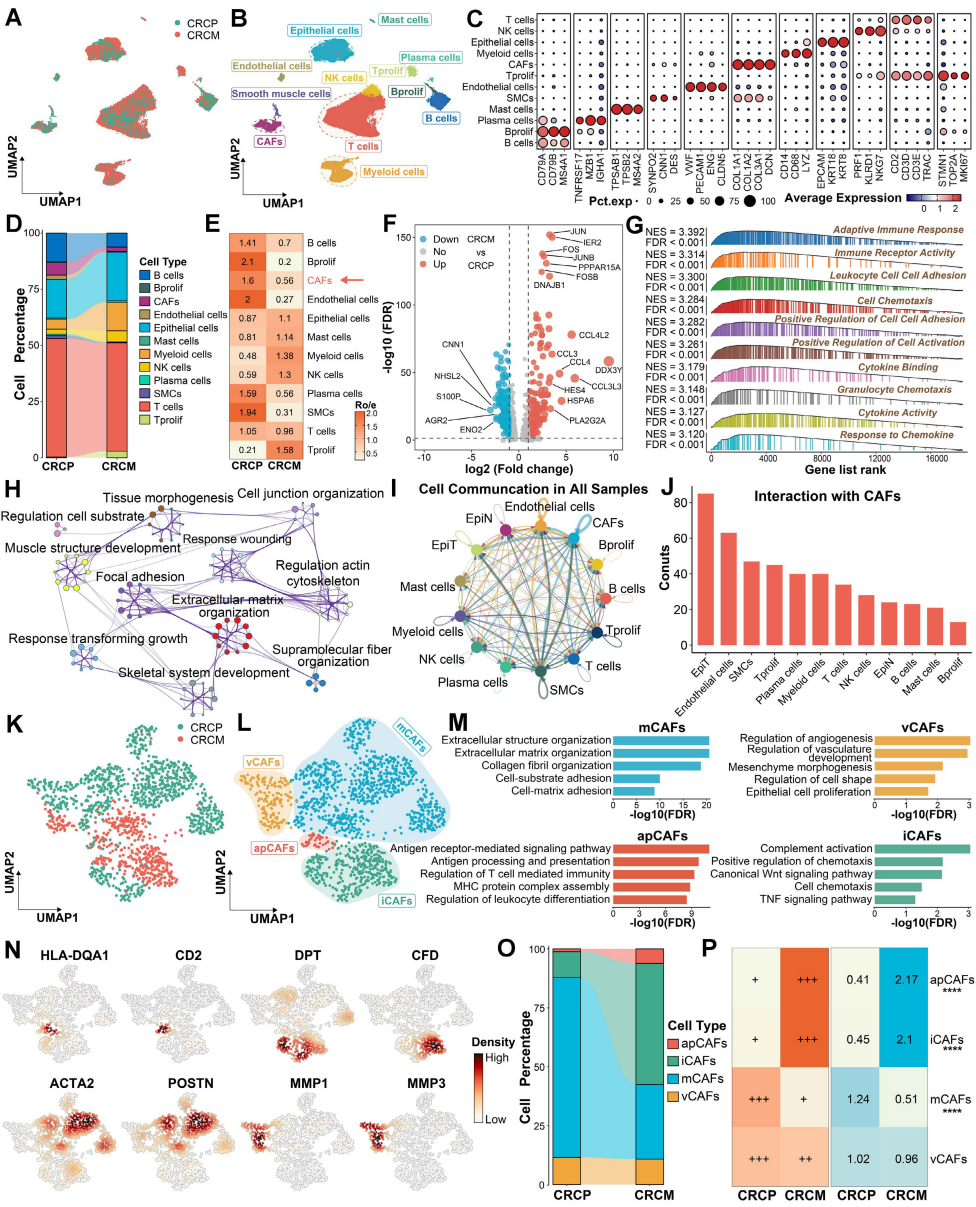
** The difference and alterations of CAFs between CRCP and CRCM.** (A-B). Dimensionality reduction and unsupervised clustering delineating the distribution of scRNA-seq data. (C). Dot plot exhibiting the expression of classical cell markers among major cell types in CRC. (D). Sankey diagram showing proportions of identified major cell populations within CRCP and CRCM. (E). Heatmap showing the Ro/e index of CAFs between CRCP and CRCM. (F). Volcano plot depicting the differentially expressed genes between CAFs from CRCP and CRCM. (G). Gene set enrichment analysis (GSEA) reveals functional characteristics of CAFs from CRCM. (H). Metascape showing the enriched pathways within CAFs from CRCP based on upregulated genes in this cell population. (I-J). CellChat infers cell-cell communication network among all cell types. (K-L). Dimensionality reduction plot delineating four CAFs subpopulations, including mCAFs, iCAFs, apCAFs, and vCAFs. (M). Functional enrichments uncover biological characteristics among four CAFs subpopulations. (N) The distribution and density of classical markers in identified four CAFs subpopulations. (O). Sankey diagram displaying proportions of identified CAF subpopulations within CRCP and CRCM. (P). Ro/e index supporting the preference of cell populations in CRCP and CRCM. ****P < 0.0001.

To investigate the difference between CRC with liver metastasis (CRCM) and without liver metastasis (CRCP), we enrolled three scRNA-seq datasets containing primary tumor sites, along with CRLM or non-CRLM outcome information. Batch effects from different patients were corrected using Harmony 17 (Figure 1A and Figure S1A). Following dimensionality reduction and unsupervised clustering, we identified 11 distinct cell types within CRC tissues (Figure 1B-1C). A comparison of the cell type proportions revealed a decrease in CAFs, which displayed lower Ro/e index 22 in CRCM compared to CRCP (Figure 1D-1E). Several molecules, including FOS, CCL3, and CCL4, were significantly elevated in CAFs from the CRCM group, promoting inflammatory chemotaxis and activation (Figure 1F). Enrichment analysis indicated that CAFs from CRCM were predominantly linked to inflammation pathways, while CAFs from CRCP were mainly involved in extracellular matrix remodeling (Figure 1G-1H and Figure S1B). Notably, CellChat analysis 21 suggested that the most prominent cell-cell communications were observed between CAFs and tumor epithelial cells (Figure 1I-1J). Recognizing the importance of CAFs in CRLM, we further explored their heterogeneity to identify distinct subpopulations. Further subcluster analysis revealed distinct CAF subpopulations, including mCAFs, iCAFs, apCAFs, and vCAFs, based on classical marker molecules and previous studies 3, 4, 8 (Figure 1K-1L). The robustness of these CAFs subpopulations was confirmed aligning with functional enrichment results, marker molecules, and signature scoring (Figure 1M-1N and Figure S1C). Compared to CRCP, the proportion of iCAFs increased, while mCAFS decreased in CRCM, with the Ro/e index 22 suggesting a preference of iCAFs in CRCM group (Figure 1O-1P). These findings underscore the potential significance of CAFs proportion changes in CRLM.

### CFD^+^ iCAFs as a key subpopulation of liver metastasis and prognosis in CRC driving factor in promoting CRLM

**Figure 2:**
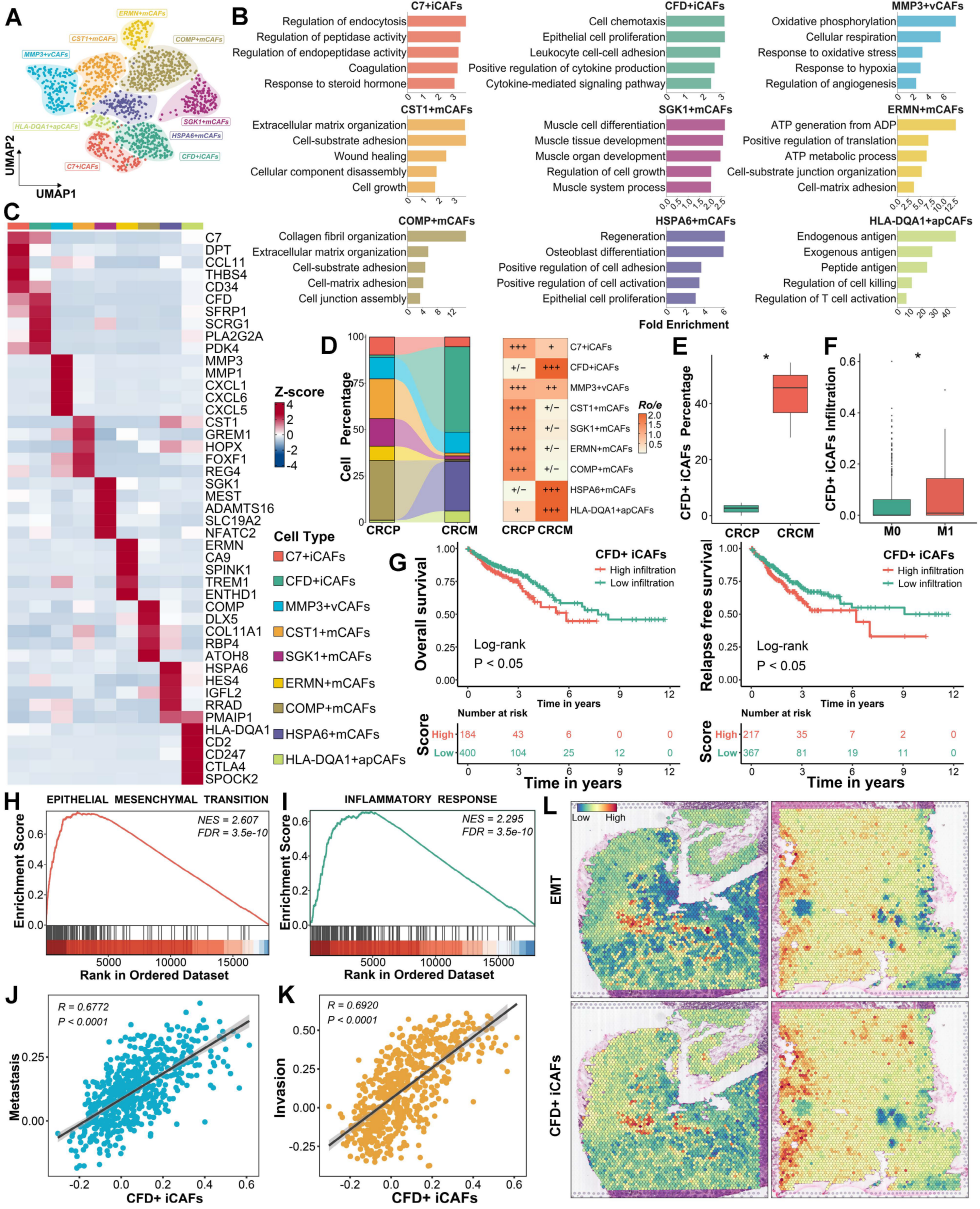
** CFD^+^ iCAFs significantly correlated with tumor EMT and metastasis.** (A). Focusing on all CAFs, dimensionality reduction and unsupervised clustering depicting the mapping of nine cell subpopulations in CRC. (B). Functional enrichment analysis uncovers specific biological pathways among different CAFs subpopulations. (C). Heatmap illustrating highly expressed genes among identified nine CAFs subpopulations in CRC. (D). Sankey diagram displaying proportions and Ro/e index supporting the preference of identified nine CAFs subpopulations. (E-F). Box plot showing the CFD^+^ iCAFs percentage in scRNA-seq data and comparing the infiltration of CFD^+^ iCAFs in TCGA transcriptomic data. (G). Kaplan-Meier survival analysis reveals the prognosis value of CFD^+^ iCAFs. (H-I). GSEA highlights the significant biological pathways in CFD^+^ iCAFs. (J-K). Correlation analysis suggests the strong links between CFD^+^ iCAFs and tumor metastasis and invasion activity, respectively. (L). Spatial transcriptomic analysis of CFD^+^ iCAFs and tumor EMT in primary CRC tissues.

To explore the role of CAFs in CRLM, nine CAF subpopulations were further identified based on distinct expression profiles and functional characteristics (Figure 2A-2C). Among these subpopulations, the proportions of CFD^+^ iCAFs, HSPA6^+^ mCAFs, and HLA-DQA1^+^ apCAFs were markedly elevated in CRCM, potentially correlating with tumor metastasis (Figure 2D and Figure S2A). Focusing on their abundance differences, CFD^+^ iCAFs showed a notably higher abundance in CRCM, which were further validated through deconvolution analysis 29 in bulk transcriptome dataset, revealing elevated infiltration in metastatic patients (Figure 2E-2F). Similarly, HSPA6^+^ mCAFs exhibited high infiltration, whereas HLA-DQA1^+^ apCAFs presented the opposite pattern, with lower infiltration observed in metastatic patients (Figure S2B-S2C). To assess their clinical implications, we then scored these cell signatures 20 and found that the higher abundance of CFD^+^ iCAFs was obviously associated with dismal prognosis (Figure 2G). The abundance of HSPA6^+^ mCAFs showed no prognostic value, while higher abundance of HLA-DQA1^+^ apCAFs was associated with a favorable prognosis (Figure S2D-S2E). Given the poor prognosis significance of CFD^+^ iCAFs, we further explored their potential function. Our results unveiled that CFD^+^ iCAFs in the TME are significantly involved in pathways related to epithelial-mesenchymal transition (EMT) and inflammatory response pathways (Figure 2H-2I). The ssGSEA analysis 20, based on the CanserSEA database 30, further quantified tumor metastasis and invasion activity, revealing a strong positive correlation with CFD^+^ iCAFs (Figure 2J-2K). Spatial transcriptomics confirmed the close connection between CFD^+^ iCAFs and EMT activity, underscoring their essential role in driving tumor metastasis (Figure 2L).

### SFRP1 secretion by CFD^+^ iCAFs correlates with poor prognosis and liver metastasis in CRC

**Figure 3:**
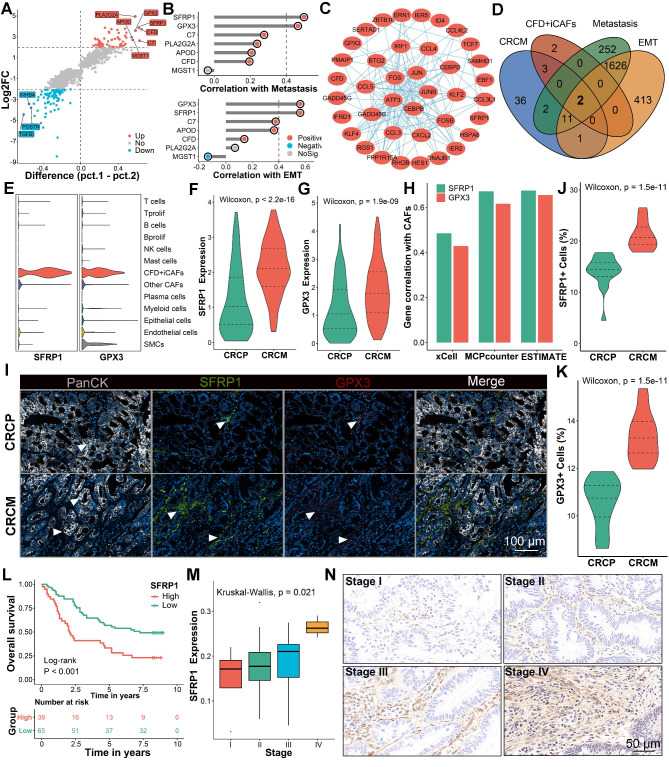
** Secretory protein SFRP1 derived from CFD^+^ iCAFs displays implications in driving CRLM.** (A). The identification of biomarkers for CFD^+^ iCAFs from CRCM, with labeled genes showing a difference of over 0.5 in two percentage categories. (B). Correlation analysis explores the links between significantly upregulated labeled genes and tumor metastasis and EMT activity. (C). The protein-protein interaction networks exhibiting interconnected relationships among the upregulated genes in CFD^+^ iCAFs from CRCM. (D). Venn diagram illustrating two overlap genes (SFRP1 and GPX3) according to upregulated genes from CRCM, feature genes from CFD^+^ iCAFs, significant positive correlation genes from EMT and metastasis with R value over 0.4. (E). Violin plots showing SFRP1 and GPX3 specifically expressed in CFD^+^ iCAFs, suggesting SFRP1 and GPX3 are derived from CFD^+^ iCAFs. (F-G). Violin plots comparing the expression of SFRP1 and GPX3 between CRCP and CRCM. (H). Using xCell, MCPcounter, and ESTIMATE tool evaluate the correlation between fibroblast abundance and expression of SFRP1 and GPX3. (I). Representative images of multiplex immunohistochemistry (mIHC) staining for SFRP1 and GPX3 in human CRCP and CRCM tissues. (J-K). Violin plots demonstrating that superior expression of SFRP1 and GPX3 in human CRCM tissues, especially for SFRP1, as assessed by mIHC staining analysis. (L-M). In a tissue microarray (TMA), Kaplan-Meier survival curves showing the association between high SFRP1 expression and poorer overall survival, alongside with advanced tumor stage. (N). Representative images of immunohistochemistry (IHC) staining for SFRP1 in CRC tissues with diverse clinical stages.

The key downstream molecules executing the functional roles of CFD^+^ iCAFs were further investigated. Starting with the identification of differentially expressed genes (DEGs), with genes showing a difference of over 0.5 in two percentage categories labeled (Figure 3A). Several of these genes, including SFRP1 and GPX3, encoded secretory proteins and were significantly upregulated in CFD^+^ iCAFs from CRCM (Figure 3A). To explore their potential roles in metastasis, correlation analysis indicated that SFRP1 and GPX3 were strongly associated with EMT and metastasis activity 30, suggesting their potential roles in promoting metastasis (Figure 3B). Protein-protein interaction (PPI) analysis further illustrated the interconnected network among the significantly upregulated genes in CFD^+^ iCAFs from CRCM (Figure 3C). SFRP1 and GPX3 were identified as key overlapping genes among the upregulated genes in CRCM, feature genes from CFD^+^ iCAFs, and genes significantly correlated with EMT and metastasis (R > 0.4) (Figure 3D). These genes were specifically derived from CFD^+^ iCAFs and highly expressed in CRCM (Figure 3E-3G). Additionally, bulk transcriptome data indicated that SFRP1 and GPX3 were positively associated with CAF abundance 31 (Figure 3H).

To validate these findings at the protein level, multiplex immunohistochemistry (mIHC) further confirmed the high expression of SFRP1 and GPX3 in human CRCM tissues, with SFRP1 demonstrating particularly prominent expression (Figure 3I-3K). Analysis of a tissue microarray (TMA) cohort revealed that high SFRP1 expression was dramatically associated with poor prognosis and advanced tumor stages in CRC patients (Figure 3L-3M). Similar findings indicated that elevated GPX3 expression was linked to dismal prognosis and was more prevalent in advanced stages (Figure S3A-S3B). Further supporting these observations, IHC analysis of human CRC tissues also confirmed high SFRP1 expression from stroma cells was positively correlated with tumor progression, underscoring its critical role in promoting metastasis (Figure 3N). Collectively, our findings suggest that SFRP1 derived from CFD^+^ iCAFs plays a pivotal role in CRLM.

### Transcription factor FOS facilitates the differentiation of CFD^+^ iCAFs and SFRP1 expression

**Figure 4:**
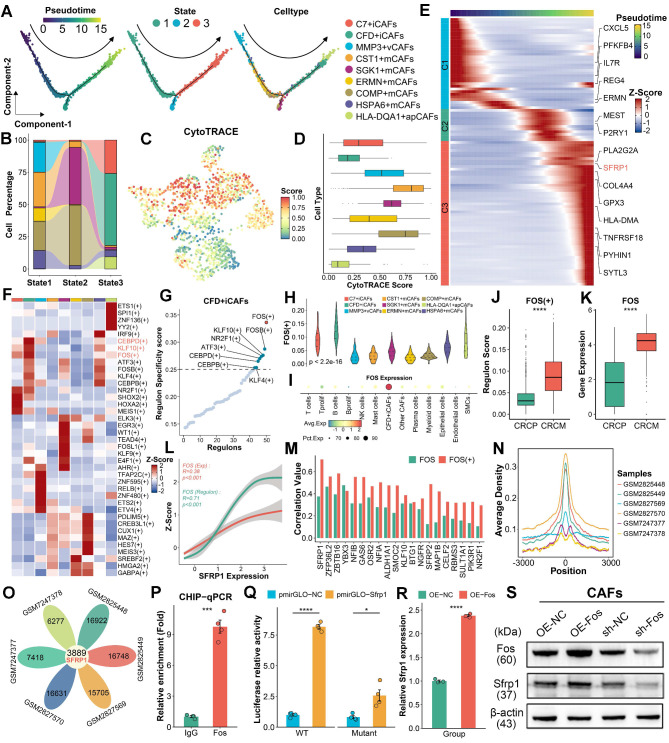
** FOS facilitates the formation of CFD^+^ iCAFs and increases SFRP1 expression.** (A-B). Pseudotime analysis highlights the differentiation of CAFs subpopulations, depicting three distinct states. (C-D). Dimensionality reduction plot displaying the distribution of CytoTRACE scores and Box plot comparing CytoTRACE scores across these nine CAFs subpopulations. (E). Heatmap illustrating the dynamic expression of key pseudotime associated genes. (F). SCENIC analysis evaluates transcription factor activity across distinct CAFs subpopulations. (G). Regulon specificity score (RSS) plot displaying FOS as the top-ranked transcription factor in CFD^+^ iCAFs. (H-I). Violin plot comparing the RSS levels of FOS across CAFs subpopulations and dot plot exhibiting FOS expression among various cell types. (J-K). Box plots comparing the RSS and expression levels of FOS between CAFs derived from CRCP and CRCM. (L). The correlation of FOS expression and transcriptional activity with SFRP1 expression. (M). The correlation of FOS expression and transcriptional activity with top 20 targeted genes expression across CAFs subpopulations. (N-O). FOS ChIP-sequencing analysis reveals its high transcriptional regulatory activity and venn diagram showing the overlap FOS targeted genes. (P). ChIP-qPCR analysis showing significant enrichment of Fos at the promoter region of Sfrp1. (Q). Luciferase reporter assay suggesting that Fos enhances the transcriptional activity of the wild-type Sfrp1 promoter relative to a mutant type. (R). qPCR analysis of Sfrp1 mRNA level in primary mouse CAFs with Fos control and overexpression. (S). Western blot analysis of Sfrp1 protein level in primary mouse CAFs with Fos knockdown or overexpression.

Given the importance of CFD^+^ iCAF in CRLM, we further explored its upstream regulatory mechanism. The pseudotime analysis 23 was performed to reveal the differentiation trajectory of CFD^+^ iCAFs in CRLM. A notable observation was that the differentiation of CAFs occurred in three distinct states, with CFD^+^ iCAFs and HLA-DQA1^+^ apCAFs mainly localized at state3 (Figure 4A-4B). CytoTRACE 24 further inferred the cellular differentiation potential, revealing that CFD^+^ iCAFs, characterized by a low score, displayed limited differentiation potential and a mature functional state (Figure 4C-4D). Additionally, unsupervised clustering identified three distinct pseudotime segments, each associated with different active progression genes, such as SFRP1 and HLA-DMA, which were activated during the late process (Figure 4E).

To identify the transcriptional regulators driving this process, the SCENIC pipeline 25 was employed to identify transcriptional regulatory networks among different CAF subpopulations, pinpointing key regulons specific to cell subgroup (Figure 4F). In CFD^+^ iCAFs, FOS was identified as a potential transcriptional regulator, as it exhibited the highest regulon specificity score (RSS) (Figure 4G). Furthermore, FOS displayed the highest RSS and expression in CFD^+^ iCAFs compared to other cell subpopulations, implying its important regulatory role (Figure 4H-4I). Notably, both regulon activity and expression of FOS were elevated in CAFs derived from CRCM (Figure 4J-4K). Correlation analysis revealed SFRP1 expression was prominently associated with FOS regulon activity and expression (Figure 4L). Among transcription factor-targeted genes, SFRP1 displayed the strongest link with FOS (Figure 4M). These results suggest that FOS regulates SFRP1 expression.

To confirm the regulatory role of FOS on SFRP1, utilizing the FOS ChIP-sequencing data, we identified the centered genomic region exhibiting high binding activity (Figure 4N). Notably, SFRP1 was a commonly targeted regulatory gene of FOS across all samples (Figure 4O). ChIP-qPCR confirmed that Fos was dramatically enriched in the promoter region of Sfrp1 (Figure 4P). Additionally, luciferase reporter assays demonstrated that Fos significantly enhanced luciferase activity of the wild-type Sfrp1 promoter (Figure 4Q). To further support the role of Fos in upregulating Sfrp1 expression, we performed knockdown and overexpression experiments in mouse CAFs. Western blot (WB) and qPCR analysis demonstrated that knockdown of Fos reduced Sfrp1 expression, while Fos overexpression increased Sfrp1 expression in CAFs (Figure 4R-4S). These findings underscore the pivotal role of FOS in regulating the differentiation of CFD^+^ iCAFs and enhancing SFRP1 expression.

### SFRP1 promotes tumor stemness and EMT activity *in vitro*

**Figure 5:**
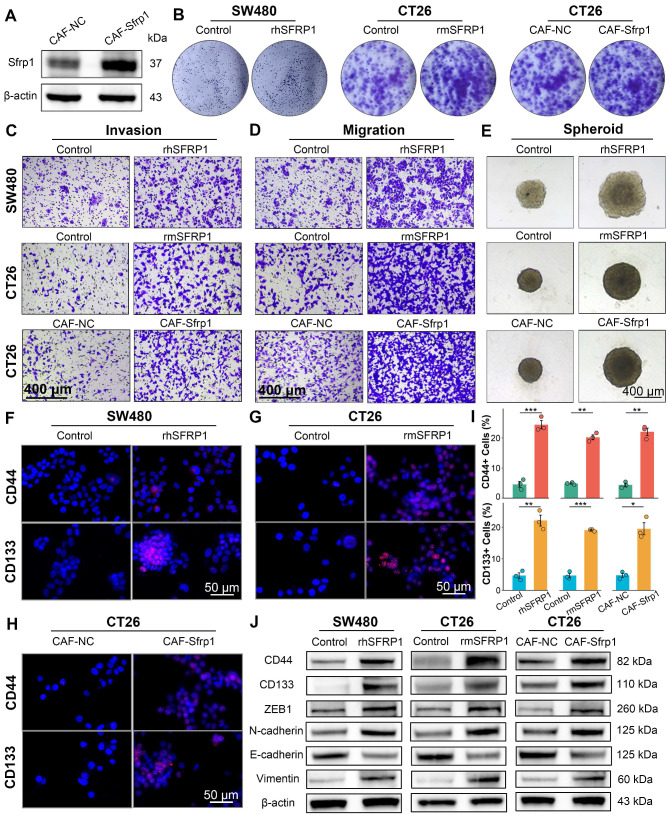
** SFRP1 performs a pro-metastatic biological role *in vitro*.** (A). Western blot analysis tests the efficiency of lentiviral transduction, confirming Sfrp1 overexpression in primary mouse CAFs. (B). Colony formation assay detecting colony number to assess proliferative capacity of CRC cells. (C). Invasion assay assessing invasive activity of CRC cells. Representative images of invasion assay. Scale bars, 400 μm. (D). Migration assay assessing migratory activity of CRC cells. Representative images of migration assay. Scale bars, 400 μm. (E). Spheroid assay reflecting stemness of CRC cells. Representative images of spheroid assay. Scale bars, 400 μm. (F-H). CD44 and CD133 staining evaluating stemness of CRC cells. Representative images of immunofluorescence. Scale bars, 50 μm. (I). Quantification of CD44 and CD133 staining and identification of positive cells ratio, comparing the effects of recombinant proteins and CAF CM on CRC cells. (J). Western blot analysis tests tumor stemness and EMT associated proteins, including CD44, CD133, ZEB1, Vimentin, N-cadherin, and E-cadherin, on recombinant proteins or CAFs CM treated CRC cells, suggesting SFRP1 promotes tumor stemness and EMT activity *in vitro*. *P < 0.05, **P < 0.01, ***P < 0.001.

Further investigation of SFRP1 was conducted using the recombinant human SFRP1 protein (rhSFRP1), recombinant mouse SFRP1 protein (rmSFRP1), and stable Sfrp1 overexpression of CAFs (CAF-Sfrp1). The rhSFRP1 and rmSFRP1 were successfully constructed and validated by SDS-PAGE and WB analyses (Figure S4A-S4B). Additionally, to establish a stable overexpression system, primary CAFs were isolated and transfected with the Sfrp1 gene and empty vector (CAF-NC), and WB verified the efficiency of lentiviral transduction (Figure 5A). To enhance the universality and reproducibility of the findings, we performed cross-species simulation by rhSFRP1, rmSFRP1, and CAF-Sfrp1. The results showed that both rhSFRP1 and rmSFRP1 treatments significantly increased colony formation ability, and the conditioned medium (CM) from CAF-Sfrp1 also enhanced colony number, indicating increased proliferative capacity (Figure 5B and Figure S4C). Invasion and migration assays revealed that tumor cells with these simulations displayed increasing invasive and migratory activities (Figure 5C-5D and Figure S4D-S4E). The spheroid formation assay suggested these treatments led to the formation of tumor spheroids with significantly larger size, more compact structures, and well-defined boundaries, indicating higher proliferative activity (Figure 5E and Figure S4F). CD44 and CD133 staining further demonstrated that these treatments enhanced the presence of tumor cells with high stemness activity (Figure 5F-5I). Collectively, these findings indicate the crucial role of SFRP1 in promoting cell proliferation, invasion, migration, and stemness. WB assays also confirmed that treatment with rhSFRP1, rmSFRP1, and CM from CAF-Sfrp1 enhanced tumor stemness and EMT activity, as evidenced by upregulation of CD44, CD133, ZEB1, Vimentin, and N-cadherin, along with the downregulation of E-cadherin (Figure 5J). Taken together, these results suggest secretory protein SFRP1-producing CAFs promotes tumor stemness and EMT activity, thereby contributing to tumor progression.

### SFRP1 facilitates tumor stemness and metastasis in preclinical models

**Figure 6:**
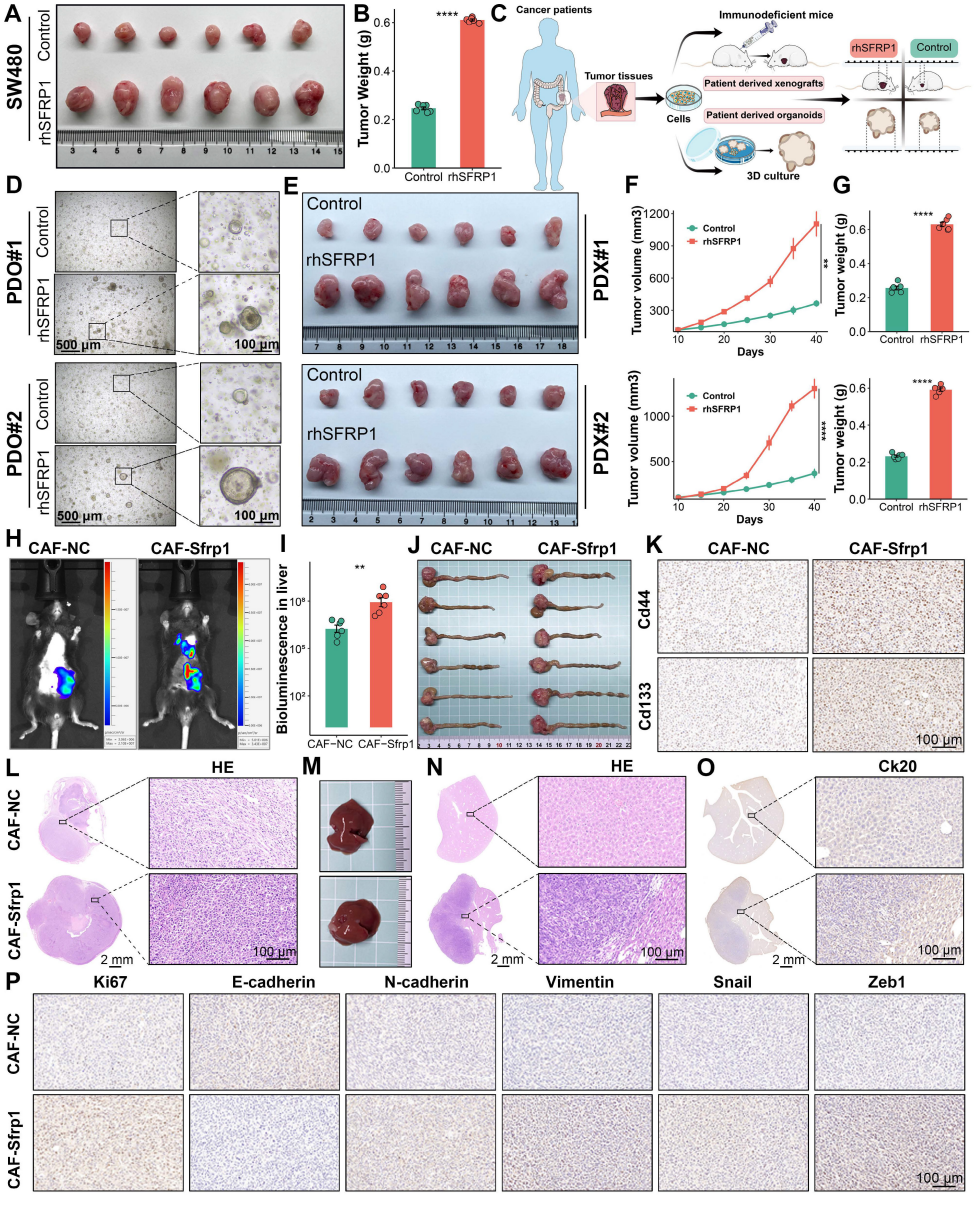
** SFRP1 promotes tumor stemness and metastasis in preclinical models.** (A). Representative images of subcutaneous xenografts model from SW480 cells. (B). Tumor weights of subcutaneous xenografts model from SW480 cells. The rhSFRP1 treatment group displayed larger tumor weight, markedly accelerating tumor growth *in vivo* setting. (C). The diagram of establishing patient derived organoids (PDOs), patient derived xenograft (PDX) models. (D). PDOs models are successfully established by two CRC patients and classified into PDO#1 and PDO#2. The rhSFRP1 treatment group display significantly larger, indicating enhanced proliferation. (E). PDX models are successfully established by two CRC patients and classified into PDX#1 and PDX#2. Representative images of PDX models from two patients treated with control rhSFRP1. (F-G) Tumor growth curves and final tumor weights of PDX models. The rhSFRP1 treatment group displays significantly larger volumes and weights compared to control group. (H). Representative bioluminescence images of orthotopic tumor metastasis models. (I). Quantification of bioluminescence in metastatic liver from orthotopic tumor metastasis models. (J). Representative images of CRC tissues form orthotopic tumor metastasis models. (K). Representative images of Cd44 and Cd133 staining of CRC tissues from orthotopic tumor metastasis models. Scale bars, 100 μm. (L-N). Representative images of hematoxylin and eosin (H&E) staining of CRC tissues and metastatic liver tissues from orthotopic tumor metastasis models. Scale bars, 2 mm and 100 μm. Gross images of metastatic liver tissues from orthotopic tumor metastasis models in CAF-Sfrp1 and CAF-NC groups. (O). Representative images of Ck20 staining of metastatic liver tissues from orthotopic tumor metastasis models. Scale bars, 2 mm and 100 μm. (P). Immunohistochemical analysis of CRC tissues from orthotopic tumor metastasis models. Scale bars, 100 μm. **P < 0.01, ****P < 0.0001.

To better elucidate the role of SFRP1 in CRLM, we employed several preclinical models, including subcutaneous tumor, patient derived organoids (PDOs), patient derived xenografts (PDXs), and orthotopic colorectal cancer metastasis models. To assess the *in vivo* effects of SFRP1 on tumor growth, subcutaneous xenograft models were constructed by injecting SW480 cells into BALB/c nude mice, followed by intratumoral injections of PBS or rhSFRP1 (Figure 6A). The rhSFRP1 treatment group displayed larger tumor size and weight, markedly accelerating tumor growth *in vivo* (Figure 6B and Figure S5A). PDOs and PDXs were generated according to the scheme and pipeline outlined in Figure 6C. Fresh CRC tumor tissues from two patients. In the PDOs models, rhSFRP1 treatment resulted in significantly larger diameter organoids, indicating enhanced proliferation (Figure 6D). Similarly, the corresponding PDXs models, generated from the same patients, showed increased tumor size and weight following rhSFRP1 treatment, suggesting enhanced proliferative activity (Figure 6E-6G).

*In vivo* fluorescence imaging revealed that the CAF-Sfrp1 treatment group exhibited stronger fluorescence signals and a broader distribution range in liver region, which notably promoted the formation of liver metastases (Figure 6H-6I). For the orthotopic tumor model, CT26 cells were pre-cultured with CAF-Sfrp1 or CAF-NC and orthotopically injected into BALB/c mice. Compared to the CAF-NC group, the CAF-Sfrp1 group significantly increased the weight of the primary CRC tumor (Figure 6J and Figure S5B). To assess tumor stemness, CD44 and CD133 staining showed stronger signals in the CAF-Sfrp1 treatment group, indicating higher stemness in tumor cells during tumor formation and progression (Figure 6K). Hematoxylin and eosin (H&E) staining of CRC tissues revealed increased cellularity and nuclear atypia in CAF-Sfrp1 group (Figure 6L). Moreover, the whole livers of CAF-Sfrp1 group developed more metastatic lesions than the CAF-NC group (Figure 6M). H&E and IHC of liver tissues also displayed severe histological changes and increased malignant marker Ck20 expression in CAF-Sfrp1 group (Figure 6N-6O). Additionally, IHC analysis of CRC tissues revealed upregulated expression of proliferation markers Ki67, as well as EMT markers N-cadherin, Vimentin, Snail, Zeb1, and N-cadherin, with a concomitant decrease in E-cadherin expression in the CAF-Sfrp1 group (Figure 6P). Together, these findings demonstrate that CAF-derived SFRP1 played a critical role in facilitating tumor stemness and metastasis in CRLM, providing valuable insights into its potential as a therapeutic target.

### SFRP1 directly interacts with FGFR2 receptor on tumor cells

**Figure 7:**
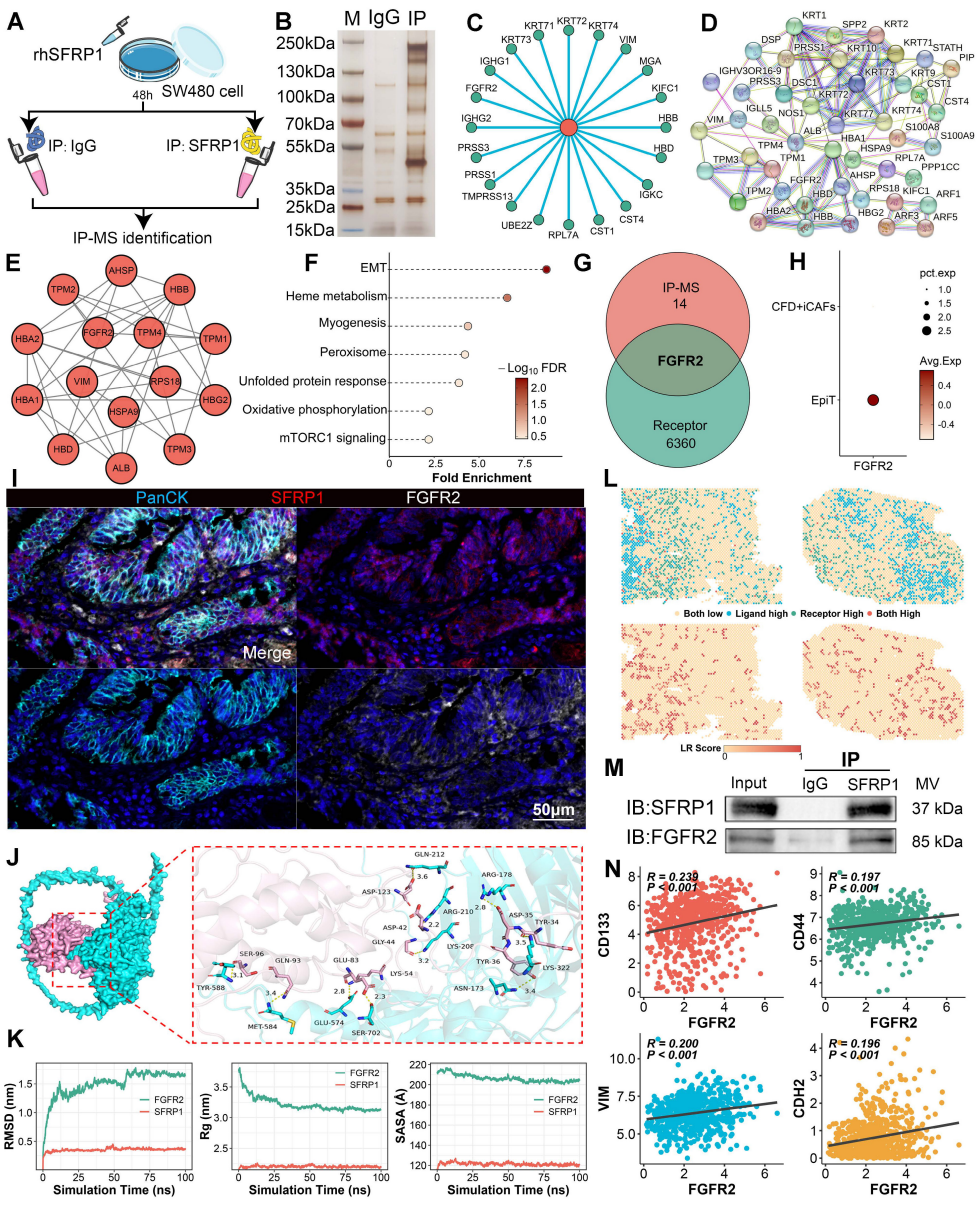
** Secretory protein SFRP1 interacts with FGFR2 receptor on tumor cells in CRC.** (A). The scheme of immunoprecipitation-mass spectrometry (IP-MS) identifying SFRP1 interacted proteins in SW480 cells mixed with rhSFRP1. (B). SDS-PAGE and silver staining exhibiting interacted protein bands in the IP group (SFRP1 antibody) compared to the IgG control group. (C). Circle plot showing top 20 proteins interacting with SFRP1 by mass spectrometry analysis. (D-E). Protein-protein interaction (PPI) networks displaying all SFRP1 interacted proteins and MCODE analysis identifying hub module of SFRP1 interacted proteins. (F). Functional enrichment analysis explores the biological pathways associated with identified SFRP1 interacted proteins, indicating activated EMT pathway. (G). Venn diagram showing overlap proteins between hub module of SFRP1 interacted proteins and cell communication associated receptors. (H). Dot plot displaying FGFR2 specifically expressed in epithelial tumor cells compared to CFD^+^ iCAFs. (I). mIHC images showing spatial proximity between high SFRP1-expressing CAFs and FGFR2-expressing tumor cells. Representative images of immunofluorescence. Scale bars, 50 μm. (J). Molecular docking and dynamics simulations displaying SFRP1-FGFR2 interaction, with structural stability. (K). Quantification of Root Mean Square Deviation (RMSD), Radius of Gyration (Rg), and Buried Surface Area (SASA) values to evaluate SFRP1-FGFR2 interaction in 100-nanosecond simulation. (L). Spatial transcriptomics reveals SFRP1 interacted with FGFR2 in primary CRC tissues. (M). Co-immunoprecipitation (CO-IP) experiment confirming the direct physical interaction between SFRP1 and FGFR2 in SW480 cells. (N). Correlation analysis demonstrates a positive association between SFRP1 expression and CD133, CD44, VIM, and CDH2 expression in TCGA transcriptomic data.

The immunoprecipitation-mass spectrometry (IP-MS) workflow was performed to investigate how SFRP1 influenced tumor cells to promote metastasis (Figure 7A). Through IP-MS, we identified and analyzed protein complexes containing SFRP1 in CRC cells. SDS-PAGE and silver staining revealed distinct protein interactions within SFRP1-associated complexes (Figure 7B). Following this, to identify specific interacting partners, mass spectrometry analysis identified the top 20 proteins interacting with SFRP1, indicating a robust network of interactions (Figure 7C). Protein-protein interaction (PPI) analysis further revealed a highly interconnected network of SFRP1-interacting proteins (Figure 7D). MCODE analysis 32 refined this network to a critical functional cluster associated with EMT activity (Figure 7E-7F), suggesting that these SFRP1 partners may play a pivotal role in EMT processes. Meanwhile, considering the apparent advantages of ligand-receptor interactions in signal transduction 33, FGFR2 emerged as the only receptor identified in this analysis (Figure 7G). FGFR2 expression was elevated in malignant epithelial cells, pointing to its potential role in tumor progression (Figure 7H). These indicated that FGFR2 may serve as a critical receptor mediating the effects of SFRP1. The mIHC was further employed to visualize the spatial relationship between SFRP1 and FGFR2 in the TME (Figure 7I). FGFR2^+^ tumor cells were observed clustering near SFRP1^+^ cells, indicating a preferential association between the two (Figure 7I).

Molecular docking and dynamics simulations explored the stability of the SFRP1-FGFR2 interaction. A 3D binding model showed specific amino acid interactions contributing to structural stability (Figure 7J). Stability analyses--Root Mean Square Deviation (RMSD), Radius of Gyration (Rg), and Buried Surface Area (SASA)--demonstrated that the SFRP1-FGFR2 interaction stabilized within a 100-nanosecond simulation (Figure 7K). Spatial transcriptomics reinforced this finding, revealing consistent SFRP1-FGFR2 interaction patterns across the TME (Figure 7L). To experimentally confirm the direct interaction, co-immunoprecipitation (Co-IP) assays further validated the direct interaction between SFRP1 and FGFR2 (Figure 7M). These results demonstrate that SFRP1 and FGFR2 form a stable ligand-receptor complex. Moreover, FGFR2 expression was significantly associated with tumor stemness and EMT markers, including CD133, CD44, VIM, and N-cadherin (Figure 7N), underscoring the role of the SFRP1-FGFR2 interaction in CRLM.

### SFRP1 activates the HIF1 pathway via FGFR2 receptor to promote stemness and metastasis

**Figure 8:**
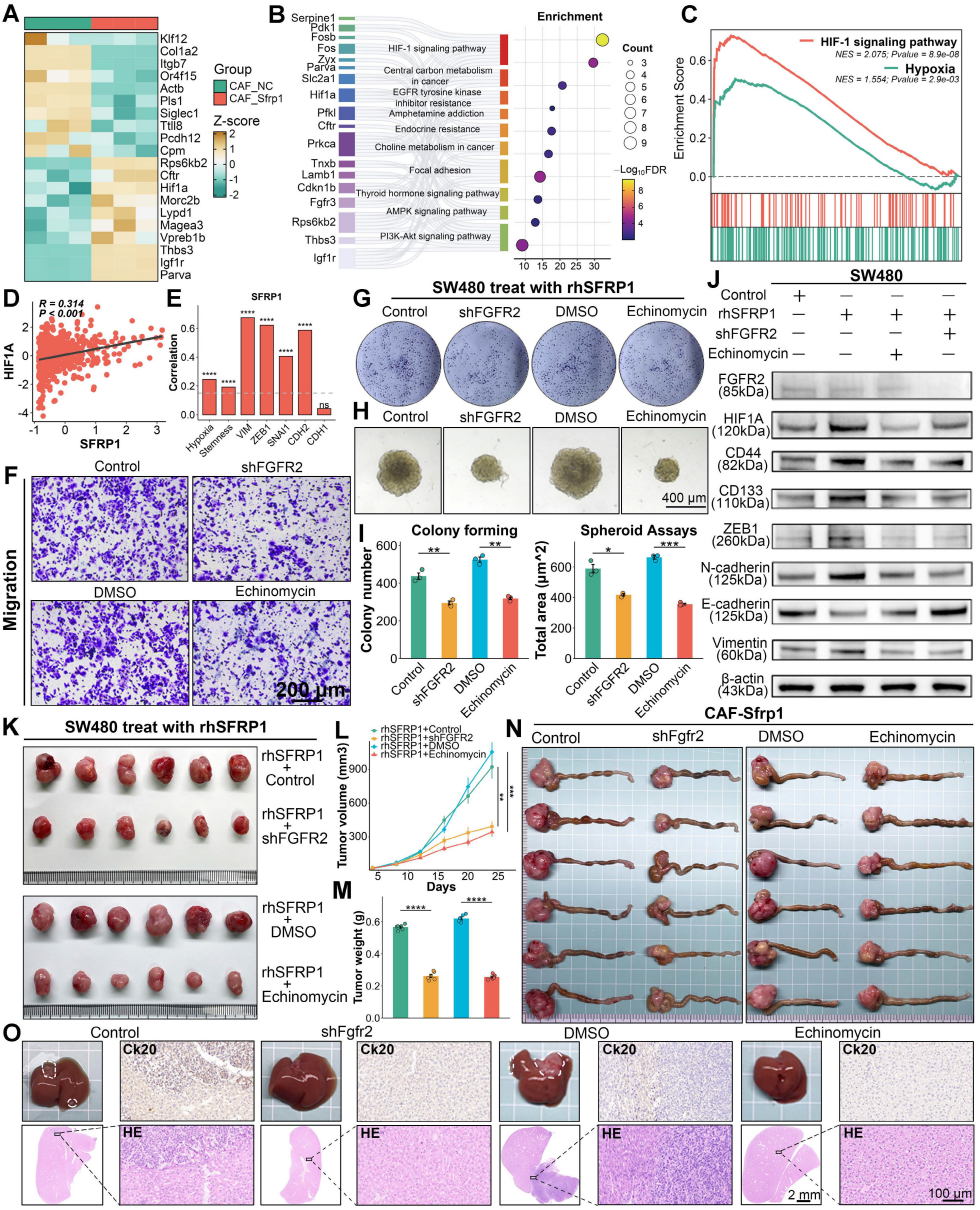
** SFRP1 interacts with FGFR2 promoting tumor stemness and metastasis through HIF1 pathway.** (A). Heatmap showing DEGs based on transcriptomic sequencing data. (B). Gene Ontology analysis of these DEGs suggests enrichment of the HIF1 signaling pathway in CRC cells pre-culture with CAF-Sfrp1. (C). GSEA analysis highlights positive association between SFRP1 and HIF1 signaling pathway as well as hypoxia signaling pathway. (D). Correlation analysis of SFRP1 expression with HIF1A expression, revealing a positive association between them in CRC samples from TCGA dataset. (E) Bar plot displaying correlations between THBS2 expression and hypoxia activity, stemness activity, and EMT associated genes in TCGA dataset. The hypoxia and stemness activities are assessed through CancerSEA dataset. (F). Migration assay assessing migratory activity across four experimental groups. Representative images of migration assay. Scale bars, 200 μm. (G). Colony formation assay detecting colony number to assess proliferative capacity across four experimental groups. (H) Spheroid assay reflecting stemness across four experimental groups. Representative images of spheroid assay. Scale bars, 400 μm. (I). Quantification of colony formation and spheroid assays results, comparing the effects of shFGFR2 and Echinomycin on SW480 CRC cells. (J). Western blot analysis of HIF1A, CD44, CD133, ZEB1, E-cadherin, N-cadherin, and Vimentin in rhSFRP1, shFGFR2, and Echinomycin treated SW480. (K). Representative images of subcutaneous xenografts models from SW480 cells across four experimental groups. (L-M). Tumor growth curves and final tumor weights of subcutaneous xenografts models. Both shFGFR2 and Echinomycin treatment significantly suppressed tumor growth even in the presence of rhSFRP1. (N). Representative images of CRC tissues from orthotopic tumor metastasis models across four experimental groups. (O). Representative images of metastatic livers with anatomical gross, Ck20 staining, and H&E staining. Scale bars, 2 mm and 100 μm. **P < 0.01, ***P < 0.001, ****P < 0.0001.

To further elucidate the downstream effects of SFRP1-FGFR2 interaction in CRLM, RNA sequencing was conducted on CRC cells pre-culture with CAF-NC or CAF-Sfrp1. Several tumor progression-associated genes, such as Hif1a and Parva, were significantly upregulated in the CAF-Sfrp1 group (Figure 8A). Functional enrichment analysis revealed that the CAF-sfrp1 group significantly enriched the HIF1 signaling pathway (Figure 8B). Consistent findings from GSEA analysis further emphasized a strong association between SFRP1 and hypoxia signaling activities (Figure 8C). Using TCGA transcriptomic data, we assessed hypoxia and stemness scores for individual patients 20, 31, corroborating significant correlations between SFRP1 expression and hypoxia, stemness, and EMT (Figure 8D-8E). These findings suggest that SFRP1 interacts with FGFR2 to activate the HIF1 pathway, promoting tumor stemness and metastasis.

To confirm this hypothesis, a series of *in vitro* experiments were conducted using four groups: empty vector transfected rhSFRP1 (Control) treated SW480 cells, rhSFRP1 (shFGFR2) treated stable FGFR2 knockdown SW480 cells, rhSFRP1 and DMSO (DMSO) treated SW480 cell, and rhSFRP1 and the HIF1 pathway inhibitor Echinomycin (Echinomycin) treated SW480 cells. Migration and invasion assays suggested that the shFGFR2 and Echinomycin groups exhibited significantly reduced invasive and migratory activities (Figure 8F and Figure S6A-S6C). Colony formation and spheroid assays indicated that the shFGFR2 and Echinomycin groups displayed reduced proliferation and stemness (Figure 8G-8I). Additionally, WB assays confirmed that rhSFRP1 treatment enhanced HIF1 signaling and was accompanied by increased expression of CD44, CD133, ZEB1, N-cadherin, and Vimentin, along with decreased E-cadherin expression (Figure 8J). These alterations were reversed in the shFGFR2 and Echinomycin groups, suggesting SFRP1 promotes tumor stemness and metastasis through FGFR2 and HIF1 pathway (Figure 8J).

The role of FGFR2 and HIF1A signaling was further validated in subcutaneous xenograft and orthotopic tumor metastasis models. Both shFGFR2 and Echinomycin treatment significantly suppressed tumor growth, leading to smaller tumor volume and weight, even in the presence of rhSFRP1 (Figure 8K-8M). In orthotopic tumor metastasis models, BALB/c mice were inoculated with either CT26 cells transfected with empty vector or stable shFgfr2 knockdown CT26 cells that all had been pre-cultured with CAF-Sfrp1. Meanwhile, CT26 cells pre-cultured with CAF-Sfrp1 were also employed to establish models, and the tumors were treated with DMSO or Echinomycin via intraperitoneal injections every two days. The shFgfr2 and Echinomycin groups displayed significantly smaller primary CRC tumors (Figure 8N and Figure S6D), as well as fewer and smaller liver metastases (Figure 8O). H&E and Ck20 staining confirmed that liver metastases in these groups exhibited reduced cellular atypia and lower malignancy (Figure 8O). These findings highlight FGFR2 and the HIF1 signaling pathway as crucial mediators in SFRP1-driven tumor metastasis.

## Discussion

Although the importance of TME in tumor metastasis is well-established 34, 35, our findings provided deeper insights into the specific involvement of CAFs in promoting CRLM. Compared with other CAF subtypes, CFD^+^ iCAFs not only exhibited immune inflammatory characteristics, but also performed closely associated with biological activities such as EMT and metastasis. Omics analysis suggested that CFD^+^ iCAFs were closely linked to poor prognosis, advanced disease stages, and liver metastasis. Trajectory analysis, ChIP assay, and luciferase reporter assay demonstrated transcription factor FOS regulated the differentiation of CFD^+^ iCAFs, promoting effector molecule SFRP1 expression. Secretory protein SFRP1 from CFD^+^ iCAFs, performed a critical role in promoting metastasis. Mechanistically, our study demonstrated that SFRP1-producing CAFs interact with cancer cells via the SFRP1-FGFR2-HIF1 signaling axis to drive both tumor stemness and metastatic potential.

Recent studies have emphasized the distinct heterogeneity and notable plasticity of CAFs in shaping the TME and influencing tumor behavior 7, 8. CAFs are not a uniform cell population performing all functions simultaneously 36, 37. These distinct CAFs with unique phenotypic characteristics perform specific biological effects. The iCAFs with secretory phenotype could secrete various cytokines or chemokines and mCAFs performed matrix remodeling, promoting tumor progression 8, 37. Although scRNA-seq had cast a revealing light on the hidden diversity of CAFs, each with unique gene expression signature and critical contributions to tumor ecosystem 8, the specific CAF subtypes drive CRLM and poor prognosis remain blurry. Our study leveraged scRNA-seq and spatial transcriptomics identified SFRP1-producing CFD^+^ iCAFs served as a pivotal subtype driving metastasis. Unlike mCAFs characterized by FAP, which mediated ECM remodeling and promoted cancer cells invasion and metastasis through TGF-β signaling 7, or apCAFs marked by HLA-DRA, which involved in modulating T cells responses via antigen presentation within the TME 38, 39, CFD^+^ iCAFs exerted their influence through a distinct manner. The secretory protein SFRP1 from CFD^+^ iCAFs promoted tumor stemness and EMT activity, thereby leading to CRLM. These insights not only expanded the functional repertoire of CAFs but also highlighted the SFRP1-producing CFD+iCAFs as pro-metastatic role in CRC.

Notably, CAFs within the TME evolve dynamically, and their plasticity is central to understanding cells fate and cells transformation 10, 40. Focusing on CAFs plasticity would contribute to decipher how these pro-metastatic CFD^+^ iCAFs arise in TME. Low secretory protein TGF-β gradient and high secretory protein IL-1 gradient regulated functional divergence of CAFs, driving the differentiation and formation of iCAF 41, 42. Additionally, TFs mediate the development of CAFs with distinct function and phenotype, promoting CAF polarization 13. Specifically, AP-1 complex plays a key role in downregulating p62, driving CAF activation 43, and FOS regulates effector gene expression, promoting pro-tumorigenic CAF activity 44. While previous studies have highlighted the role of FOS in CAF activation, the detailed mechanisms of cell transformation remained unclear. Our findings demonstrate that FOS is highly expressed in CFD^+^ iCAFs, driving the differentiation and formation of CFD^+^ iCAFs and contributing to the production of effector molecule SFRP1.

Emerging research underscores the importance of secretory proteins as reservoirs of disease biomarkers and potential therapeutic targets, highlighting their transformative impact on disease diagnosis and treatment strategies 45, 46. For example, recent findings demonstrate that secretory proteins such as Cathepsin F and Fibulin-1 serve as novel circulating diagnostic biomarkers, enabling early detection and clinical monitoring of brain metastases in lung cancer patients 47. Similarly, mCAFs secrete exosomal PWAR6, which alters glutamine availability to promote CRC liver metastasis, with PWAR6 showing promise as a therapeutic target in preclinical models 48. In our study, we identified SFRP1, a secretory protein derived from CFD^+^ iCAFs, as a key promoter of CRLM. High SFRP1 expression was associated with poor prognosis and advanced stages, suggesting its potential as both a predictive biomarker and a therapeutic target. Furthermore, the regulation of the promoter of SFRP1 by SOX2 has been shown to drive CRC cell migration and invasion 49. Other SFRP family members, such as SFRP2, have been implicated in promoting angiogenesis and metastasis when secreted by aged fibroblasts 50. Our findings provided additional clarity on the role of the SFRP family in tumor metastasis. SFRP1 interacts with the FGFR2 receptor, activating downstream HIF1 signaling pathways to enhance tumor stemness and EMT activity, thereby accelerating tumor metastasis. Notably, knockdown of FGFR2 or inhibition of the HIF1 pathway reduced liver metastasis in CRC. These findings provide new insights into the role of SFRP family members in tumor metastasis and highlighted the potential of SFRP1 as a therapeutic target.

While our study highlights the pivotal role of CFD^+^ iCAFs and SFRP1 in promoting CRLM, it also raises important questions about the broader impact of these cells and molecules within the TME. The TME is characterized by complex interactions among various cell types. For example, CXCL3^+^ macrophages interacting with CXCR2^+^ CAFs can drive a transition to myofibroblastic CAFs, ultimately promoting immune evasion and metastasis 51. Such intricate crosstalk suggests the need for further research into whether CFD^+^ iCAFs and SFRP1 similarly influence other TME components or contribute to metastasis through additional pathways. Moreover, although we demonstrate that FOS upregulates SFRP1, the upstream factors driving FOS expression remain unclear and warrant future investigation. Additionally, while our findings suggest that SFRP1 holds promise as a biomarker, its clinical utility must be rigorously validated through larger cohorts and robust clinical trials to determine its reliability and therapeutic potential.

## Conclusions

In summary, we identify CFD^+^ iCAFs as a key stromal component closely linked to dismal prognosis, advanced disease stages, and liver metastasis. FOS drives the differentiation and formation of CFD^+^ iCAFs, promoting effector molecule SFRP1 expression. SFRP1-producing CAFs interacts with tumor cells via SFRP1-FGFR2-HIF1 axis, facilitating tumor stemness and EMT. Our findings emphasize the pro-metastatic role of CFD^+^ iCAFs in the TME and highlight SFRP1 as a potential therapeutic target in CRC.

## Supplementary Material

Supplementary methods and figures.

## Abbreviations

CAFs: Cancer-associated fibroblasts
CRC: colorectal cancer
CRLM: colorectal cancer liver metastasis
scRNA-seq: single-cell RNA sequencing
EMT: epithelial-mesenchymal transition
PDOs: patient-derived organoids
PDXs: patient-derived xenografts
rhSFRP1: recombinant human SFRP1
rmSFRP1: recombinant mouse SFRP1
DEG: differentially expressed gene
H&E: hematoxylin and eosin

## References

[1] Li R, Liu X, Huang X, Zhang D, Chen Z, Zhang J (2024). Single-cell transcriptomic analysis deciphers heterogenous cancer stem-like cells in colorectal cancer and their organ-specific metastasis. Gut.

[2] Canellas-Socias A, Sancho E, Batlle E (2024). Mechanisms of metastatic colorectal cancer. Nat Rev Gastroenterol Hepatol.

[3] Cords L, de Souza N, Bodenmiller B (2024). Classifying cancer-associated fibroblasts-The good, the bad, and the target. Cancer Cell.

[4] Liu Y, Zhang X, Gu W, Su H, Wang X, Wang X (2024). Unlocking the crucial role of cancer-associated fibroblasts in tumor metastasis: Mechanisms and therapeutic prospects. J Adv Res.

[5] Fang Z, Meng Q, Xu J, Wang W, Zhang B, Liu J (2023). Signaling pathways in cancer-associated fibroblasts: recent advances and future perspectives. Cancer Commun (Lond).

[6] Cords L, Tietscher S, Anzeneder T, Langwieder C, Rees M, de Souza N (2023). Cancer-associated fibroblast classification in single-cell and spatial proteomics data. Nat Commun.

[7] Chhabra Y, Weeraratna AT (2023). Fibroblasts in cancer: Unity in heterogeneity. Cell.

[8] Lavie D, Ben-Shmuel A, Erez N, Scherz-Shouval R (2022). Cancer-associated fibroblasts in the single-cell era. Nat Cancer.

[9] Li Y, Zheng H, Luo Y, Lin Y, An M, Kong Y (2023). An HGF-dependent positive feedback loop between bladder cancer cells and fibroblasts mediates lymphangiogenesis and lymphatic metastasis. Cancer Commun (Lond).

[10] Zhang F, Ma Y, Li D, Wei J, Chen K, Zhang E (2024). Cancer associated fibroblasts and metabolic reprogramming: unraveling the intricate crosstalk in tumor evolution. J Hematol Oncol.

[11] Ma C, Yang C, Peng A, Sun T, Ji X, Mi J (2023). Pan-cancer spatially resolved single-cell analysis reveals the crosstalk between cancer-associated fibroblasts and tumor microenvironment. Mol Cancer.

[12] Luo H, Xia X, Huang LB, An H, Cao M, Kim GD (2022). Pan-cancer single-cell analysis reveals the heterogeneity and plasticity of cancer-associated fibroblasts in the tumor microenvironment. Nat Commun.

[13] Zhang X, Zhang M, Sun H, Wang X, Wang X, Sheng W (2025). The role of transcription factors in the crosstalk between cancer-associated fibroblasts and tumor cells. J Adv Res.

[14] Lee HO, Hong Y, Etlioglu HE, Cho YB, Pomella V, Van den Bosch B (2020). Lineage-dependent gene expression programs influence the immune landscape of colorectal cancer. Nat Genet.

[15] Che LH, Liu JW, Huo JP, Luo R, Xu RM, He C (2021). A single-cell atlas of liver metastases of colorectal cancer reveals reprogramming of the tumor microenvironment in response to preoperative chemotherapy. Cell Discov.

[16] Khaliq AM, Erdogan C, Kurt Z, Turgut SS, Grunvald MW, Rand T (2022). Refining colorectal cancer classification and clinical stratification through a single-cell atlas. Genome Biol.

[17] Tran HTN, Ang KS, Chevrier M, Zhang X, Lee NYS, Goh M (2020). A benchmark of batch-effect correction methods for single-cell RNA sequencing data. Genome Biol.

[18] Patel AP, Tirosh I, Trombetta JJ, Shalek AK, Gillespie SM, Wakimoto H (2014). Single-cell RNA-seq highlights intratumoral heterogeneity in primary glioblastoma. Science.

[19] Subramanian A, Tamayo P, Mootha VK, Mukherjee S, Ebert BL, Gillette MA (2005). Gene set enrichment analysis: a knowledge-based approach for interpreting genome-wide expression profiles. Proc Natl Acad Sci U S A.

[20] Liu L, Liu Z, Gao J, Liu X, Weng S, Guo C (2022). CD8+ T cell trajectory subtypes decode tumor heterogeneity and provide treatment recommendations for hepatocellular carcinoma. Front Immunol.

[21] Jin S, Guerrero-Juarez CF, Zhang L, Chang I, Ramos R, Kuan CH (2021). Inference and analysis of cell-cell communication using CellChat. Nat Commun.

[22] Zhang L, Yu X, Zheng L, Zhang Y, Li Y, Fang Q (2018). Lineage tracking reveals dynamic relationships of T cells in colorectal cancer. Nature.

[23] Trapnell C, Cacchiarelli D, Grimsby J, Pokharel P, Li S, Morse M (2014). The dynamics and regulators of cell fate decisions are revealed by pseudotemporal ordering of single cells. Nat Biotechnol.

[24] Gulati GS, Sikandar SS, Wesche DJ, Manjunath A, Bharadwaj A, Berger MJ (2020). Single-cell transcriptional diversity is a hallmark of developmental potential. Science.

[25] Aibar S, Gonzalez-Blas CB, Moerman T, Huynh-Thu VA, Imrichova H, Hulselmans G (2017). SCENIC: single-cell regulatory network inference and clustering. Nat Methods.

[26] Suo S, Zhu Q, Saadatpour A, Fei L, Guo G, Yuan GC (2018). Revealing the Critical Regulators of Cell Identity in the Mouse Cell Atlas. Cell Rep.

[27] Yu G, Wang LG, He QY (2015). ChIPseeker: an R/Bioconductor package for ChIP peak annotation, comparison and visualization. Bioinformatics.

[28] Liu Q, Hsu CY, Shyr Y (2022). Scalable and model-free detection of spatial patterns and colocalization. Genome Res.

[29] Newman AM, Steen CB, Liu CL, Gentles AJ, Chaudhuri AA, Scherer F (2019). Determining cell type abundance and expression from bulk tissues with digital cytometry. Nat Biotechnol.

[30] Yuan H, Yan M, Zhang G, Liu W, Deng C, Liao G (2019). CancerSEA: a cancer single-cell state atlas. Nucleic Acids Res.

[31] Zeng D, Ye Z, Shen R, Yu G, Wu J, Xiong Y (2021). IOBR: Multi-Omics Immuno-Oncology Biological Research to Decode Tumor Microenvironment and Signatures. Front Immunol.

[32] Xiong Y, Liu X, Jiang L, Hao T, Wang Y, Li T (2024). Inhibition of ferroptosis reverses heart failure with preserved ejection fraction in mice. J Transl Med.

[33] Armingol E, Officer A, Harismendy O, Lewis NE (2021). Deciphering cell-cell interactions and communication from gene expression. Nat Rev Genet.

[34] Liu Z, Chen J, Ren Y, Liu S, Ba Y, Zuo A (2024). Multi-stage mechanisms of tumor metastasis and therapeutic strategies. Signal Transduct Target Ther.

[35] Haynes NM, Chadwick TB, Parker BS (2024). The complexity of immune evasion mechanisms throughout the metastatic cascade. Nat Immunol.

[36] Feng B, Wu J, Shen B, Jiang F, Feng J (2022). Cancer-associated fibroblasts and resistance to anticancer therapies: status, mechanisms, and countermeasures. Cancer Cell Int.

[37] Biffi G, Tuveson DA (2021). Diversity and Biology of Cancer-Associated Fibroblasts. Physiol Rev.

[38] Harryvan TJ, Visser M, de Bruin L, Plug L, Griffioen L, Mulder A (2022). Enhanced antigen cross-presentation in human colorectal cancer-associated fibroblasts through upregulation of the lysosomal protease cathepsin S. J Immunother Cancer.

[39] Gao Y, Li J, Cheng W, Diao T, Liu H, Bo Y (2024). Cross-tissue human fibroblast atlas reveals myofibroblast subtypes with distinct roles in immune modulation. Cancer Cell.

[40] Tang PC, Chung JY, Xue VW, Xiao J, Meng XM, Huang XR (2022). Smad3 Promotes Cancer-Associated Fibroblasts Generation via Macrophage-Myofibroblast Transition. Adv Sci (Weinh).

[41] Biffi G, Oni TE, Spielman B, Hao Y, Elyada E, Park Y (2019). IL1-Induced JAK/STAT Signaling Is Antagonized by TGFbeta to Shape CAF Heterogeneity in Pancreatic Ductal Adenocarcinoma. Cancer Discov.

[42] Sahai E, Astsaturov I, Cukierman E, DeNardo DG, Egeblad M, Evans RM (2020). A framework for advancing our understanding of cancer-associated fibroblasts. Nat Rev Cancer.

[43] Linares JF, Cid-Diaz T, Duran A, Osrodek M, Martinez-Ordonez A, Reina-Campos M (2022). The lactate-NAD(+) axis activates cancer-associated fibroblasts by downregulating p62. Cell Rep.

[44] Mazzeo L, Ghosh S, Di Cicco E, Isma J, Tavernari D, Samarkina A (2024). ANKRD1 is a mesenchymal-specific driver of cancer-associated fibroblast activation bridging androgen receptor loss to AP-1 activation. Nat Commun.

[45] Robinson JL, Feizi A, Uhlen M, Nielsen J (2019). A Systematic Investigation of the Malignant Functions and Diagnostic Potential of the Cancer Secretome. Cell Rep.

[46] Sternberg C, Raigel M, Limberger T, Trachtova K, Schlederer M, Lindner D (2024). Cell-autonomous IL6ST activation suppresses prostate cancer development via STAT3/ARF/p53-driven senescence and confers an immune-active tumor microenvironment. Mol Cancer.

[47] Wei S, Liu W, Xu M, Qin H, Liu C, Zhang R (2022). Cathepsin F and Fibulin-1 as novel diagnostic biomarkers for brain metastasis of non-small cell lung cancer. Br J Cancer.

[48] Fang H, Dai W, Gu R, Zhang Y, Li J, Luo W (2024). myCAF-derived exosomal PWAR6 accelerates CRC liver metastasis via altering glutamine availability and NK cell function in the tumor microenvironment. J Hematol Oncol.

[49] Kasashima H, Duran A, Martinez-Ordonez A, Nakanishi Y, Kinoshita H, Linares JF (2021). Stromal SOX2 Upregulation Promotes Tumorigenesis through the Generation of a SFRP1/2-Expressing Cancer-Associated Fibroblast Population. Dev Cell.

[50] Kaur A, Webster MR, Marchbank K, Behera R, Ndoye A, Kugel CH 3rd (2016). sFRP2 in the aged microenvironment drives melanoma metastasis and therapy resistance. Nature.

[51] Sun X, He X, Zhang Y, Hosaka K, Andersson P, Wu J (2022). Inflammatory cell-derived CXCL3 promotes pancreatic cancer metastasis through a novel myofibroblast-hijacked cancer escape mechanism. Gut.

