# Supplementary material for: FOS-driven inflammatory CAFs promote colorectal cancer liver metastasis via the SFRP1-FGFR2-HIF1 axis
Source: Theranostics. 2025 Mar 21;15(10):4593–613. doi: 10.7150/thno.111625 (PMC11984394; doi:10.7150/thno.111625)
Supplement: Supplementary file 1 — Supplementary methods and figures. [file thnov15p4593s1.pdf]

Preparation of recombinant protein

The cDNA sequences for human SFRP1 (hSFRP1) and mouse SFRP1 (mSFRP1) were obtained from GenBank (NM_003012.5 and NM_013834.3). The plasmids pQE-80L/hSFRP1 and pQE-80L/mSFRP1 were synthesized by Sangon Biotech (Shanghai, China). Recombinant human SFRP1 (rhSFRP1) and recombinant mouse SFRP1 (rmSFRP1) were expressed in Escherichia coli BL21(DE3) strains, following induction with 0.5 mM isopropyl β-D-1-thiogalactopyranoside (IPTG) at 37 °C for 7 h. The recombinant proteins were purified using nickel affinity chromatography. The successful expression of rhSFRP1 and rmSFRP1 was confirmed by SDS-PAGE and Western blot analysis.

RNA isolation, reverse transcription, and qPCR

Total RNA was extracted using TRIzon reagent (CWbiotech, Jiangsu, China), and cDNA was synthesized with the HiFiScript cDNA Synthesis Kit (CWbiotech, Jiangsu, China). Quantitative real-time PCR (qPCR) was performed on a LineGene 9600 Plus Real-Time PCR System (Bioer, Hangzhou, China) using RapidStart Universal SYBR Green qPCR Mix (Monad, Suzhou, China). The relative mRNA levels were determined by the cycle threshold (Ct) method, normalized to GAPDH, and analyzed using the 2^−ΔΔCt method.

Multiplex immunohistochemistry

Tissue slides were initially deparaffinized and rehydrated, followed by antigen retrieval using microwave treatment. Endogenous peroxidase activity and nonspecific binding sites were blocked. Primary antibodies were then applied, followed by secondary antibodies conjugated to HRP polymers from the AlphaTSA fluorescent staining kit (Alpha X Bio, Beijing, China). Tyramide signal amplification (TSA) with fluorophores Opal followed for 10 min. After that, antigen retrieval step was repeated to eliminate any background signal before proceeding to subsequent labeling cycles. After multiple rounds of labeling, nuclear counterstaining was conducted with DAPI to complete the staining procedure. Color images were captured using the ZEISS AXIOSCAN slide scanning system (ZEISS, Oberkochen, Germany).

Molecular docking and dynamics simulations

To investigate the interaction between SFRP1 and FGFR2 proteins, molecular docking and dynamics simulations were performed. Using the results of molecular docking as the initial conformation of the crystal, AmberTools23 was used to calculate the BCC charge of the ligand. AmberTools23 was used to build the ligand-protein complex simulation system. A box was built around the complex, with a minimum distance of 1.0 nm between the edge of the box and the protein-ligand complex. TIP3P water was added to the box, along with 3 Na^+ to neutralize the system's charge. The Amber 14SB force field was applied to the protein, solvent, and balancing ions, while the GAFF force field was applied to the ligand. Parmed was used to generate compatible run files for Gromacs 2023. Gromacs 2023 was used for system simulation. First, the system was energy-minimized using the steepest descent method, reaching the lowest energy in 1205 steps. Then, 100 ps of NVT system equilibration and NPT system equilibration were performed separately. Finally, a 100 ns molecular dynamics simulation was conducted using the equilibrated results. The Gromacs trajconv module was used for preliminary processing of the simulation trajectory, including handling periodic boundary conditions and aligning the protein alpha carbon atoms to eliminate the translation and rotation of the protein-ligand complex system during the simulation. The processed trajectory was analyzed using MDAnalysis.

Bulk transcriptome data

The RNA sequencing cohort with completed clinical information, TCGA-CRC (n = 585), was derived from The Cancer Genome Atlas database. The data was converted into transcripts per million (TPM) style and further transformed into log2 (TPM+1) format. To further assess cell infiltration abundances, Xcell, MCPcounter, and ESTIMATE algorithms encapsulated in *IOBR* R package [1] were employed to deconvolute the proportions of fibroblast. Using CIBERSORTx [2] resource, the gene expression data from scRNA-seq was enrolled to estimate the abundances of various cell types in TCGA-CRC.

RNA sequencing

The cell line RNA sequencing was conducted on three samples each of CRC cells pre-cultured with CAF-NC and CAF-Sfrp1. The cell line RNA sequencing was conducted

on three samples each of CRC cells pre-cultured with CAF-NC and CAF-Sfrp1. Total RNA was extracted using Trizol reagent (Invitrogen, USA) and its integrity assessed using the RNA Nano 6000 Assay Kit on the Bioanalyzer 2100 system (Agilent Technologies, CA, USA). The mRNA was then purified from the total RNA using poly-T oligonucleotide-coated magnetic beads, followed by fragmentation in a first-strand synthesis reaction buffer (5×) containing divalent cations at elevated temperature. Subsequently, first and second strand cDNA synthesis was completed, and the remaining overhangs were converted into blunt ends by exonuclease/polymerase activity. To preferentially select cDNA fragments ranging from 370-420 bp in length, the library fragments were purified through the AMPure XP system (Beckman Coulter, Beverly, USA). Based on suitable fragments, PCR amplification was performed and further purified. Finally, the library was sequenced using Agilent platform. The DEGs were identified by DESeq2 with a significant threshold of P value < 0.05 and fold change > = 2.

References:

1. Zeng D, Ye Z, Shen R, Yu G, Wu J, Xiong Y, et al. IOBR: Multi-Omics Immuno-Oncology Biological Research to Decode Tumor Microenvironment and Signatures. Front Immunol. 2021; 12: 687975.

2. Newman AM, Steen CB, Liu CL, Gentles AJ, Chaudhuri AA, Scherer F, et al. Determining cell type abundance and expression from bulk tissues with digital cytometry. Nat Biotechnol. 2019; 37: 773-82.

Supplementary Figures:

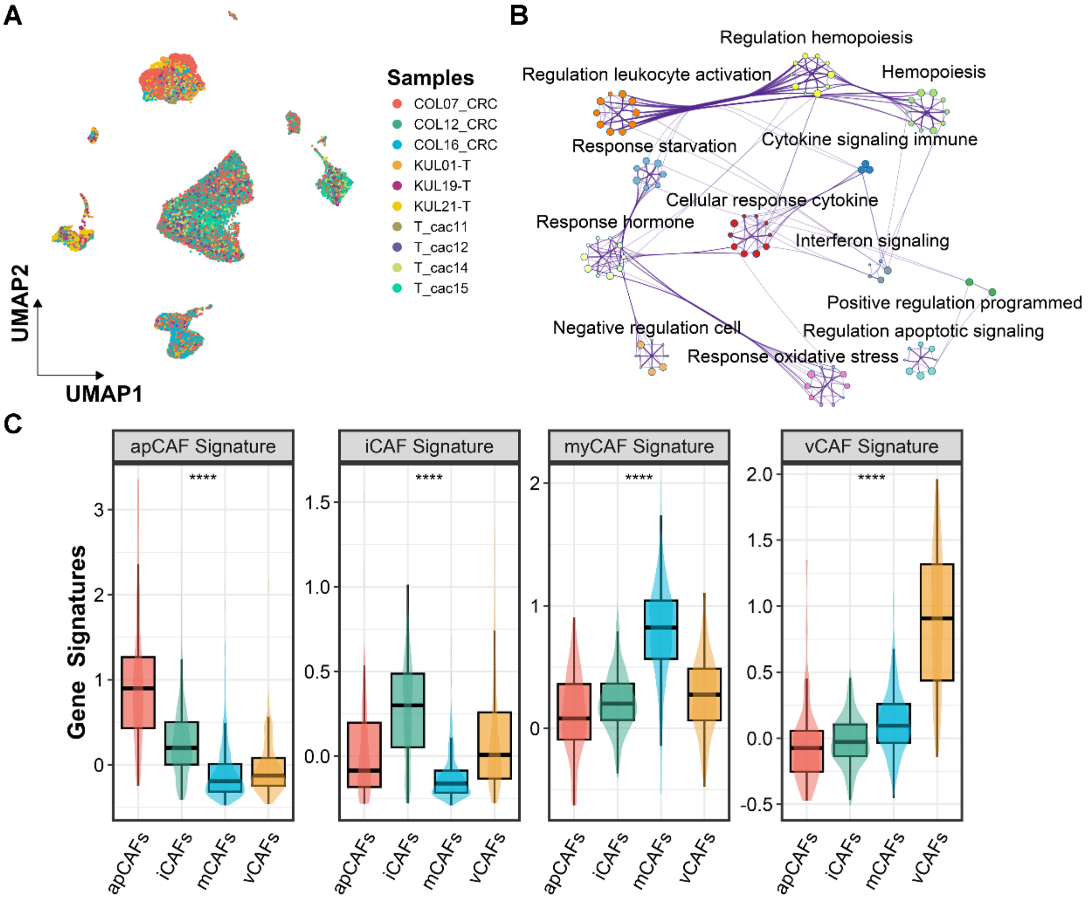

Figure S1. Clustering and identification of cell subpopulations.

(A). Dimensionality reduction showing the distribution of cell clustering from different samples processing with Harmony approach.

(B). Metascape depicting the enriched pathways within CAFs from CRCM based on upregulated genes in this cell population.

(C). Box plots comparing the signature scores calculated by AddModuleScore across four CAFs subpopulations, including mCAFs, iCAFs, apCAFs, and vCAFs. ****P < 0.0001.

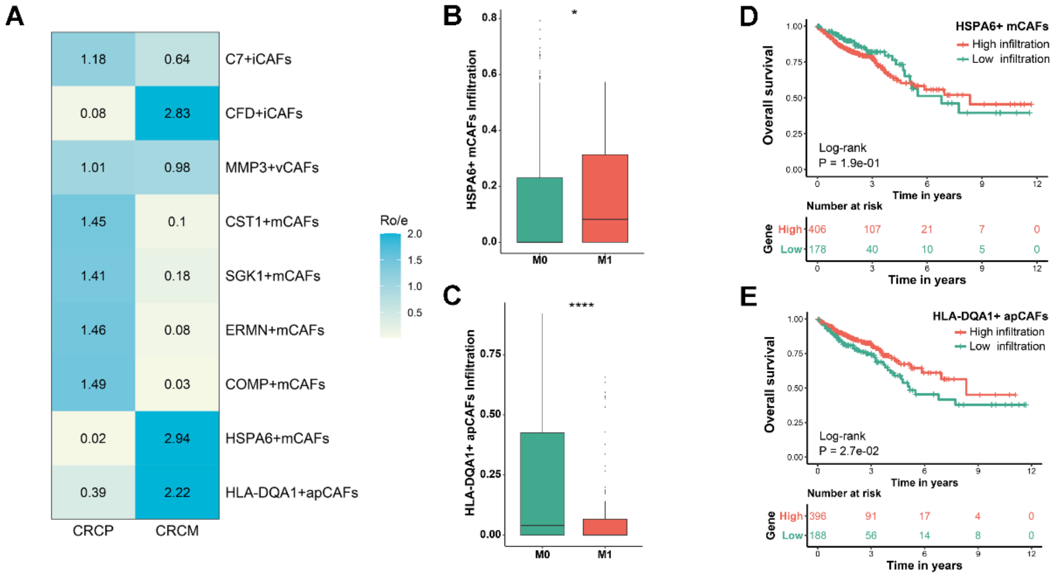

Figure S2. Prognostic and metastatic value of HSPA6^{+} mCAFs and HLA-DQA1^{+} apCAFs infiltration.

(A). The distribution of Ro/e index among different CAFs types, suggesting CFD^{+} iCAFs, HSPA6^{+} mCAFs, and HLA-DQA1^{+} apCAFs potentially associated with tumor metastasis.

(B). Box plot comparing the infiltration of HSPA6^{+} mCAFs between M0 clinical stage and M1 clinical stage in TCGA transcriptomic data.

(C). Box plot comparing the infiltration of HLA-DQA1^{+} apCAFs between M0 clinical stage and M1 clinical stage in TCGA transcriptomic data.

(D). Kaplan-Meier survival analysis suggests HSPA6^{+} mCAFs infiltration is not a prognostic indicator in overall survival in CRC patients.

(E). Kaplan-Meier survival analysis reveals the association between high HLA-DQA1^{+} apCAFs infiltration and favorable overall survival in CRC patients. *P < 0.05, ****P < 0.0001.

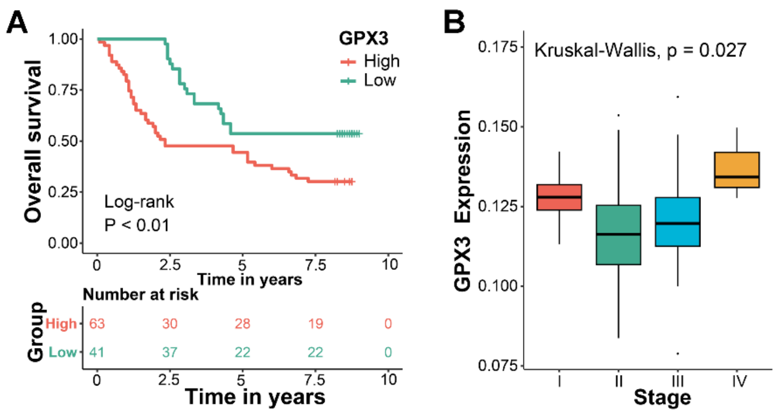

Figure S3. The implication of GPX3 in prognosis and clinical stage.

(A). Kaplan-Meier survival curves showing the association between high GPX3 expression and poorer overall survival, assessed by a tissue microarray cohort.

(B). Box plot displaying patients with high GPX3 expression are prone to advanced tumor stage in CRC.

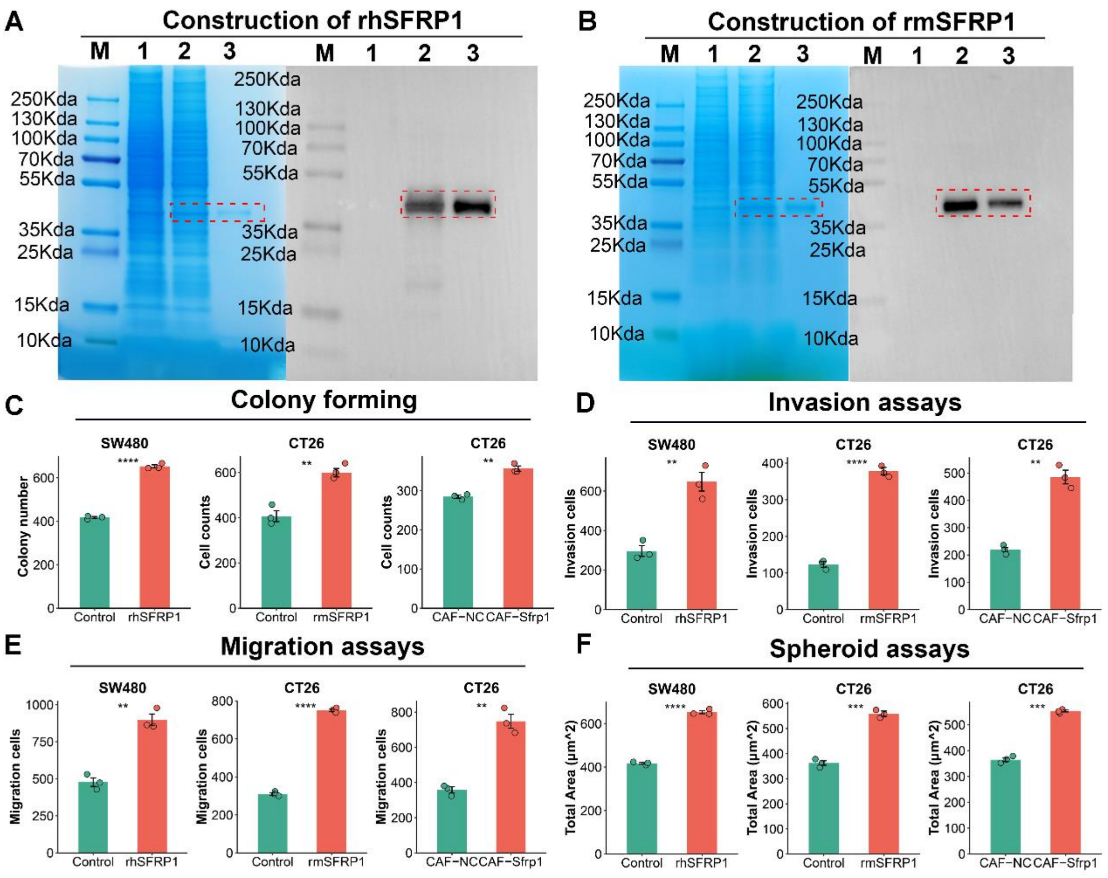

Figure S4. The construction of recombinant proteins and quantification of cell experiments.

(A). The construction of human recombinant protein SFRP1 (rhSFRP1). SDS-PAGE and Western blot confirming rhSFRP1 expression.

(B). The construction of mouse recombinant protein SFRP1 (rmSFRP1). SDS-PAGE and Western blot confirming rmSFRP1 expression.

(C). Quantification of colony formation assay results, comparing the effects of CRC cells treated with recombinant proteins (rhSFRP1 and rmSFRP1) or conditioned medium (CM) from CAFs.

(D). Quantification of invasion assay results, comparing the effects of CRC cells treated with rhSFRP1, rmSFRP1, and CM from CAFs.

(E). Quantification of migration assay results, comparing the effects of CRC cells treated with rhSFRP1, rmSFRP1, and CM from CAFs.

(F). Quantification of spheroid assay results, comparing the effects of CRC cells treated with rhSFRP1, rmSFRP1, and CM from CAFs. **P < 0.01, ***P < 0.001, ****P < 0.0001.

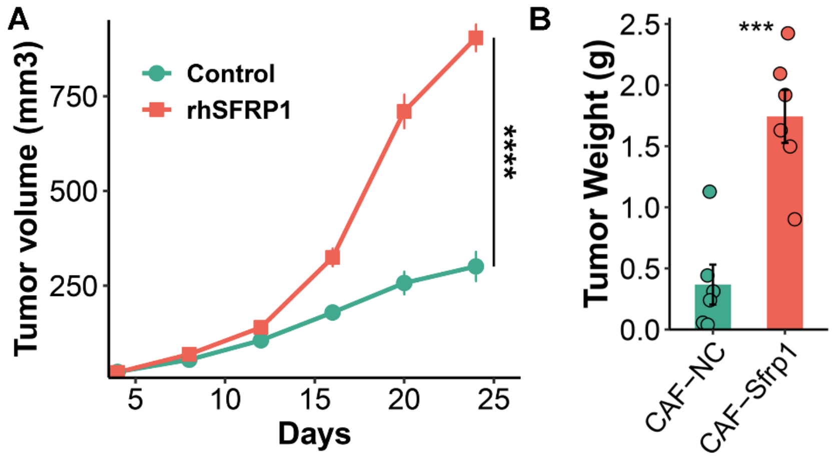

Figure S5. The tumor volume of subcutaneous xenografts models and tumor weight of orthotopic metastasis tumor models.

(A). Tumor growth curves of subcutaneous xenografts model from SW480 cells treated with control and rhSFRP1.

(B). Tumor final tumor weights of orthotopic metastasis tumor model form CT26 cells pre-cultured with primary mouse CAFs with stable overexpression of Sfrp1 (CAF-

Sfrp1) and CAFs transfected with empty vector (CAF-NC). ***P < 0.001, ****P < 0.0001.

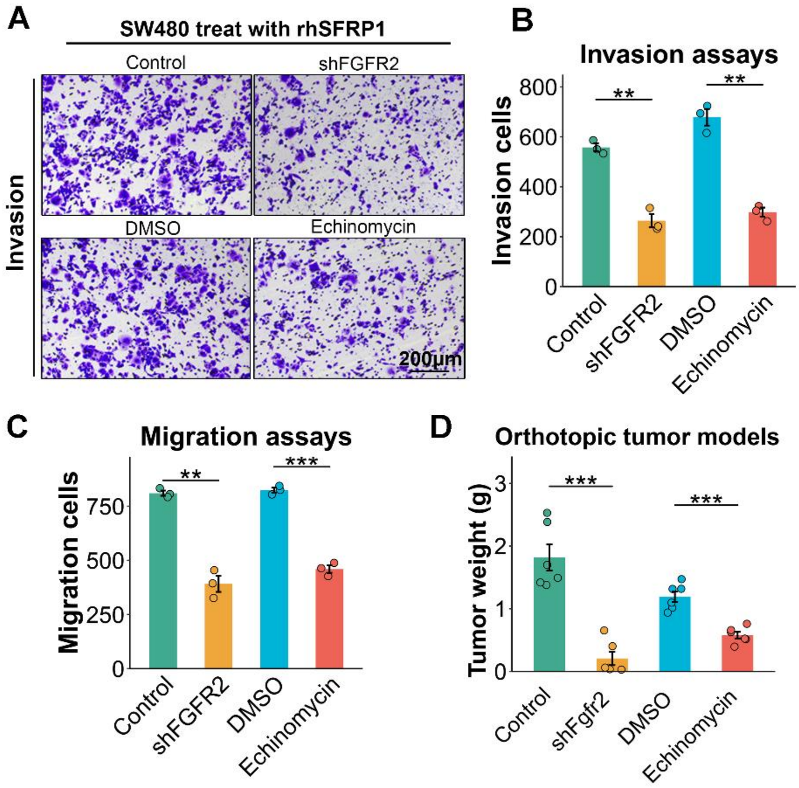

Figure S6. Evaluating the role of FGFR2 and HIF1 pathway in CRC progression. (A). Invasion assay assessing invasive activity across four experimental groups, including empty vector transfected SW480 cells treated with rhSFRP1 (Control), stable FGFR2 knockdown SW480 cells treated with rhSFRP1 (shFGFR2), SW480 cells treated with DMSO and rhSFRP1 (DSMO), and SW480 cells treated with HIF1 pathway inhibitor Echinomycin and rhSFRP1 (Echinomycin). Representative images of invasion assay. Scale bars, 200 μm.

(B). Quantification of invasion assay results, comparing the effects of shFGFR2 and Echinomycin on SW480 CRC cells.

(C). Quantification of migration assay results, comparing the effects of shFGFR2 and Echinomycin on SW480 CRC cells.

(D). Final tumor weights of orthotopic transplantation tumor models. Both shFgfr2 and Echinomycin treatment significantly suppressed tumor progression even in the presence of CAF-Sfrp1. **P < 0.01, ***P < 0.001.
